# Experimental Realization of Zenneck Type Wave-based Non-Radiative, Non-Coupled Wireless Power Transmission

**DOI:** 10.1038/s41598-020-57554-1

**Published:** 2020-01-22

**Authors:** Sai Kiran Oruganti, Feifei Liu, Dipra Paul, Jun Liu, Jagannath Malik, Ke Feng, Haksun Kim, Yuming Liang, Thomas Thundat, Franklin Bien

**Affiliations:** 1 Ulsan National Institute of Science and Technology, Ulsan, Republic of Korea; 2 Jiangxi University of Science and Technology, Ganzhou, China; 3 University at Buffalo, New York, USA

**Keywords:** Applied physics, Aerospace engineering, Electrical and electronic engineering, Energy infrastructure

## Abstract

A decade ago, non-radiative wireless power transmission re-emerged as a promising alternative to deliver electrical power to devices where a physical wiring proved impracticable. However, conventional “coupling-based” approaches face performance issues when multiple devices are involved, as they are restricted by factors like coupling and external environments. Zenneck waves are excited at interfaces, like surface plasmons and have the potential to deliver electrical power to devices placed on a conducting surface. Here, we demonstrate, efficient and long range delivery of electrical power by exciting non-radiative waves over metal surfaces to multiple loads. Our modeling and simulation using Maxwell’s equation with proper boundary conditions shows Zenneck type behavior for the excited waves and are in excellent agreement with experimental results. In conclusion, we physically realize a radically different class of power transfer system, based on a wave, whose existence has been fiercely debated for over a century.

## Introduction

In 2007, coupled WPT re-emerged as an alternative to deliver electrical power to systems where physical wiring is difficult or dangerous^1,2^. Since then, a number of notable articles appeared^3–5^. However, these were improvements or at the best variations of the coupled WPT systems originally proposed in^2^.

All the existing WPT systems (Inductive, magnetic resonance and capacitive; far field systems not included) rely on critical coupling between coils of the transmitter and receiver for efficient delivery of power^2–7^. The resonance conditions are easily affected by the external factors^6–8^. It has also been well understood that the need for a critical coupling leads to peak splitting phenomena for multiple resonant devices^7^. This causes efficiency degradation and hence, are unsuitable for emerging fields such as, internet of things (IoT) and dynamic charging of electrical vehicles. Therefore, parity time circuits method was proposed to resolve the issue of dynamic wireless charging^6^. Unfortunately, we will continue to face these limitations due to our reliance on critical coupling between the transmitter and receiver^8^.

A non-radiating *wave-based* wireless power transfer system would be a desirable candidate to solve some of these issues. Quite a few wave based systems in the *μ*-wave regime have appeared over the years. A detailed literature survey of these systems has been carried out in the Supplementary Material. Also, WPT systems saw the usage of magneto-inductive planar waveguide^9^. This kind of WPT system utilizes the concept of meta-materials and generation of standing waves. Presumably, this is the meta-material equivalent of the quarter-wave Tesla transformer.

We wish to draw the attention to Zenneck wave (Sommerfeld-Zenneck wave), which resides at the metal-air interface, akin to surface plasmons (SP) and surface waves (SW)^10,11^. All these three classes of interface waves are near-field phenomenon^12^. While SP and surface wave (SW) have been widely researched areas in optical physics and metasurfaces, they are relatively less studied in the microwave regime^12–15^. Likewise, much research around ZW is focused on the communications and geophysics applications^13,16,17^. Unfortunately, ZW has been surrounded by the controversies pertaining to their physical existence^14,15,18^. The bulk of the controversy arose from the alleged “sign error” committed by Sommerfeld in 1909^14,15^. Some authors have shown feasibility of such waves by recreating the critical Seneca lake experiment to debunk the Sommerfeld sign error myth^19^. However, articles like these lack scientific rigor^19^, this further brings disorderliness to the existing controversy.

Quite literally, one does not find any study on the utilization of ZW for non-radiative power transfer. Recently in 2014 and 2017 Sarkar et al, have taken great pains to clarify the confusions arising due to the definitions of SW, SP and ZW through their mathematically rigorous articles^14,15^. The properties exhibited by ZW’s are like SW and SP, with certain differences. All these three physical phenomena are transverse magnetic (TM) modes and exhibit evanescent field decay away from the metal-air or metal-dielectric or conductive-dielectric interface. Unlike SW, the ZW come into existence as a result of zero of the TM reflection coefficient. SP come into existence at the quasi-particle levels. Whereas, ZW propagate in the form of localized charge oscillations. Just like SW and SP, when ZW are excited on metal surfaces, the net flow of current is zero. The Brewster angle of incidence in case of ZW is frequency independent. Therefore, the attenuation of ZW waves is also frequency independent and the attenuation rate is slow in the transverse direction^14,15^. They sink into a lossy dielectric media, as mathematically demonstrated by Barlow and Cullens in their classic article^20^. This sinking phenomenon was later experimentally demonstrated in the articles^16,21^.

Here we demonstrate the physical realization of a ZW non-radiative power transmission using the arrangement of a planar ground backed impedance (GBI) surface and a half wave helical transformer at radio frequency (RF). The GBI structure establishes a TM wave across the metal surface. Whereas, the half wave helical transformer drives the voltage across the GBI terminals. The helical transformer is like the telsa transformer (Supplementary Information). However, unlike the tesla transformer it does not generate standing waves. It was earlier theorized that an infinite vertical aperture is needed to excite a Zenneck wave and hence it was not physically realizable^22^. In our results we demonstrate that, although it is not possible to excite a pure ZW, however, waves with strong ZW like properties can certainly be excited. Thus bypassing the problem of infinite vertical aperture. We also demonstrate that unlike the coupled non-radiative wireless power transmission systems, the presence of leaky metal shields does not affect the power transmission efficiency^2,23^. Moreover, we demonstrate uniform power delivery to multiple receiving units with meaningful efficiency by theory and experiment, as we eliminate the frequency peak splitting issue altogether^7^. We also demonstrate by arriving at the Eq. 1, that equi-phases of ZW waves tilt backwards in the air, at the metal-air interface^10,11^. Thus, reminiscent with the title of the article by Jeon *et.al*.^17^. This article implies that there is a link between SP and ZW’s at metal-air interface.

While efficient transmission of non-radiative, wireless power over long distances using earth as a conductor is far from practical realization, it may be possible to utilize already existing metal structures to send guided mode waves for powering electrical devices^1,24,25^. There exist many practical scenarios consisting of metallic infrastructures, such as nuclear plants, railway tracks, space ships, steel building structures, pipelines, etc. Practical applications include powering Internet of things (IoT) devices, charging for -marine vessels, smart manufacturing floors, and secured shipping containers^24–26^.

## Results

**Figure 1:**
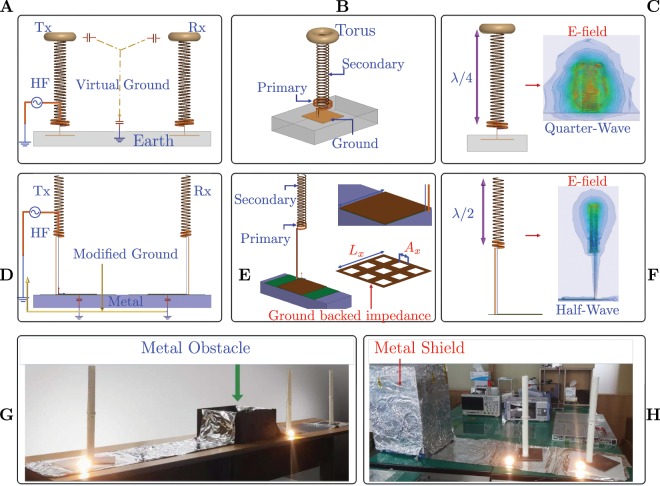
Concept of the proposed Zenneck wave system. (**A**) In Tesla transformer, grounding is an extremely critical factor. Both the transmitter and receiver are grounded to the earth ground. (**B**) Tesla transformer. (**C**) E-field buildup and standing waves. (**D**) Approach followed in this study based on half wave helical transformer. The GBI resonator sets up a TM-Mode wave. Grounding is done through the metal, which in turn pulls the reference potential of the metal to the grid ground. (**E**) Primary and Secondary coils of the proposed system. (**F**) E-Field buildup. (**G**) Power transfer across metal obstacle. (**H**) Transmitter is shielded in a leaky metal shield.

Please note, the experimental setup is described in the section 1 of the Supplementary Material. The key concept of this study is presented in Fig. 1, the detailed analytical model and solution is presented in the Supplementary Material (under section: analytical formalism). The Fig. 1A–C shows the mechanism of Tesla transformer based wireless power transfer system^1^. The primary coil consists of low number of turns, while the secondary has large number of turns (quarter-wave). One end of the secondary is left freely suspended in the air. Sometimes, a toroid is attached to the free end of the secondary to restrict the electric field buildup to prevent discharges. The primary and secondary coils on both the transmission and receiving end share the common ground, as shown in Fig. 1A–C. The generator, which operates as a high frequency AC source, is also grounded to the grid, which is in-turn grounded to the earth^1^.

### Approach followed in this study

The Fig. 1D–F shows the schematic diagram of the system to excite zenneck waves at metal-air interface. Apart from exciting TM- waves using the GBI resonators at the metal-air interface, we use two critical concepts of Tesla transformer, namely- half wave helical coil (Tesla transformer uses a quarter wave coil), in the secondary to build high potential differences across the terminals of the resonator and grounding the coils to the grid ground, capacitively via the metal. This pulls the reference potential of the metal to the same level as the grid ground. Thus, metal is transformed into a neutral entity^26^.

### Half wave helical coil

It is well known that a quarter-wave open ended helical coil, when mounted over a planar metal acts as a radiating antenna^27^. Notable application-vehicle mounted antennas, where the metal body provides a natural ground for the helical coil loaded antenna. Self resonance frequency of helical coils is well studied, the resonance frequency is active at *λ*/2 conductor length^27^. In order to prevent radiation a half wave helical coil was used and the pitch between the coils was carefully chosen in order to avoid the generation of standing waves and radiation. In this frequency zone, the lumped elements can not provide the necessary electrical length. In the Supplementary Material, the section “Electrical Length” describes the approach followed to address the above parameters in this study, in details.

### Zenneck wave type power transfer mechanism

**Figure 2:**
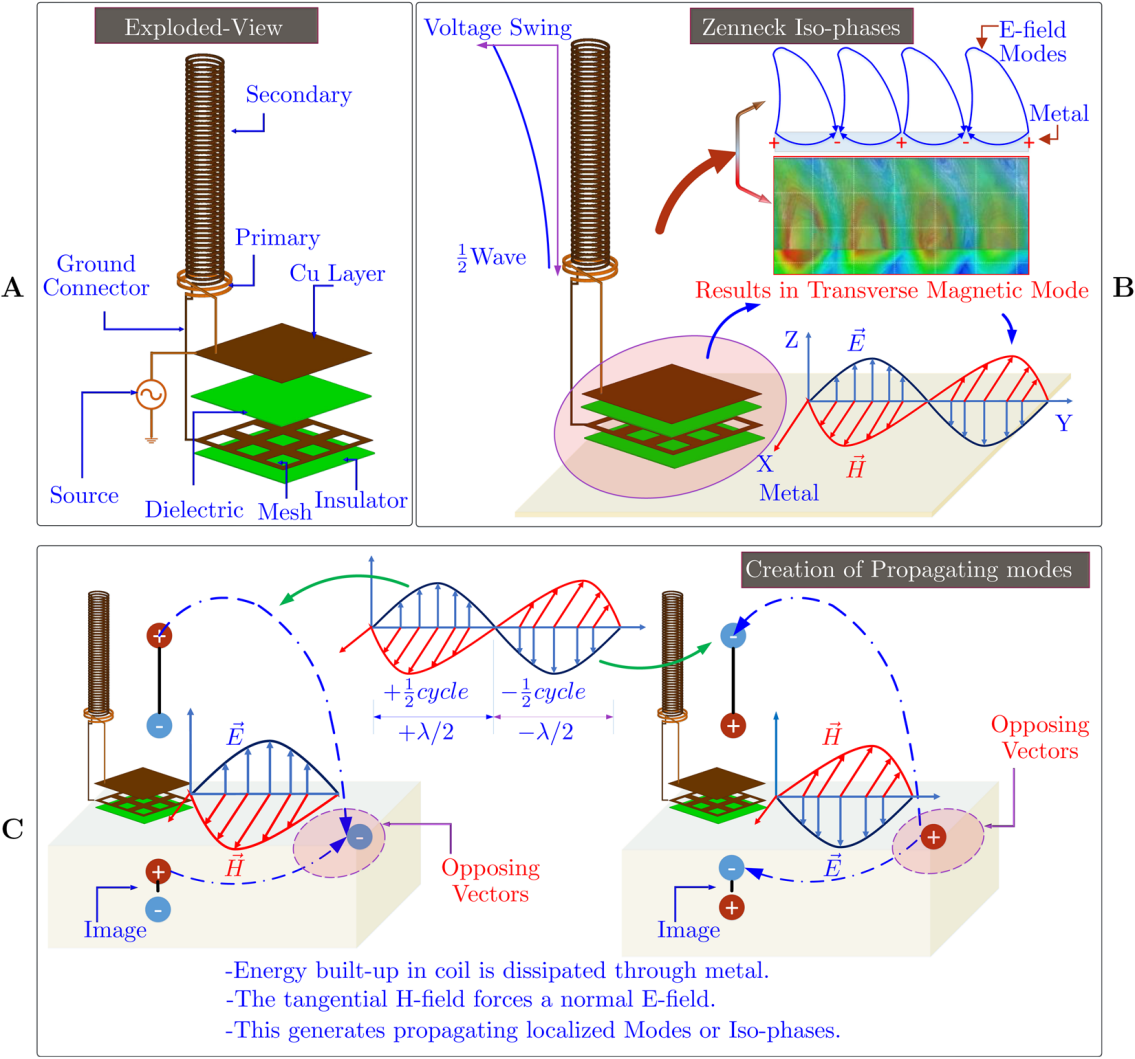
Concept of the proposed Zenneck wave system. (**A**) Details of the system in this study. (**B**) Field mechanism of the proposed system. (**C**) The mechanism of energy buildup, the E-field forms a forward and backward vector meeting at the interface (modes). The TM-wave forces the propagation of the modes.

The Fig. 2A shows the exploded view of the construction of the ZW resonator system undertaken in this study and the Fig. 2B,C shows the field mechanism. A high frequency AC source feeds power into the resonator system when placed in proximity of the metal surface. The primary coil being low turn carries maximum current, whereas a very high voltage is built up in the open ended secondary coil. The energy built-up in the secondary coil is forced to dissipate through metal as the half wave helical coils are poor radiators^27^. The E-field from the top of the coil terminates at the metal and a counter field line originates from the image formed in the metal, both meeting at the metal-air interface. This is similar to charge or field mechanisms in a lightening strike, the forward stroke due to charges in the cumulonimbus^28^. Whereas, the backward stroke is formed in the ground, both meeting at the air-ground interface^28^. Likewise, the GBI structure forces a TM-wave to be setup on the metal surface, thus facilitating a propagation of the modes formed by the non-radiating coil over metal, every half cycle of the sinusoidal high frequency source.

In the Supplementary Material (section 3) Sommerfeld analytical model is listed, the controversy surrounding the evaluation of the integral is mentioned. Finally, a commentary on the placement of pole as per the permissible Riemann sheet on the complex plane, in the case of metals is provided.

In the case of the proposed method, likelihood of the waves falling in the category of SP, SW and surface plasmon polariton (SPP) is eliminated when one considers the following conditions:

- SW: Corrugated metal structures are needed to increase the refractive index in order to excite SW’s. Or an air-dielectric-metal (three layer) interface is needed. Alternatively, inductive surface impedance is needed to excite SW^12,13,20,29^.
- SPP and SP: Can not be excited at flat metal-air interfaces, without total internal reflection. Other methods of SPP excitation is based on grooves and near-field highly focused optical beams^12,13,20,29^.

### Relation between SP and ZW

Interestingly, articles have appeared on relationship between SP and ZW. Most notably the article by Jeon *et al*., where they mention in the title THz Zenneck surface wave and in brackets THz surface plasmon on metal sheets^17^. This arises from the fact that the *pole* and *zero* of the reflection coefficient ($R(\Lambda )$) in the Sommerfeld integral (equation S25 to S27 in the Supplementary Material) coinciding on the permissible Riemann sheet^14^. Sarkar *et al*., further note that the ZW attenuation rate along the interface is frequency independent, while attenuation rate in SP is frequency dependent^14^.

### Equi-phases

The Fig. 2B, shows the iso-phases or equi-phases generated due to the localized field oscillations on the metal-air interface. The phase velocity of the wave in the metal is faster than the free space, hence a backward tilt with an angle *ϕ* is observed, in accordance with^10^. The angle of tilt has been arrived in this work from the 1907’s article of Zenneck, which satisfy the Maxwell’s boundary conditions:

1 $${\phi }_{0}={{\sin }}^{-1}[{(\sqrt{\frac{\nu \sigma {\varepsilon }_{0}}{{\sigma }^{2}+{\nu }^{2}{({\varepsilon }^{\text{'}}-j{\varepsilon }^{\text{'}\text{'}})}^{2}}})}^{-1}]$$

where, *v* = 2*π*/*λ*; *σ* is conductivity; complex permitivitty $\varepsilon ^{\prime} -j\varepsilon ^{\prime\prime}$; and free-space permitivitty *ε*_0_. The corresponding *ϕ*_0_ = 90 − *ϕ*, this was also mentioned in^20^. The derivation of the above equation is listed in the Supplementary Material (S28–S31). In case of metal-air interfaces the quantity *ϕ*_0_ becomes negative and hence a backward tilt. On the otherhand, for air-lossy dielectric this angle is a positive quantity and hence a forward tilt. For more details, see Fig. S14 in the Supplementary Material.

### Sinking of Equi-phases

Likewise, the iso-phases in the Fig. 2B, undergo a forward tilt and subsequent sinking when they encounter a lossy dielectric^16,20,21^.

### Hallmark of Zenneck waves

**Figure 3:**
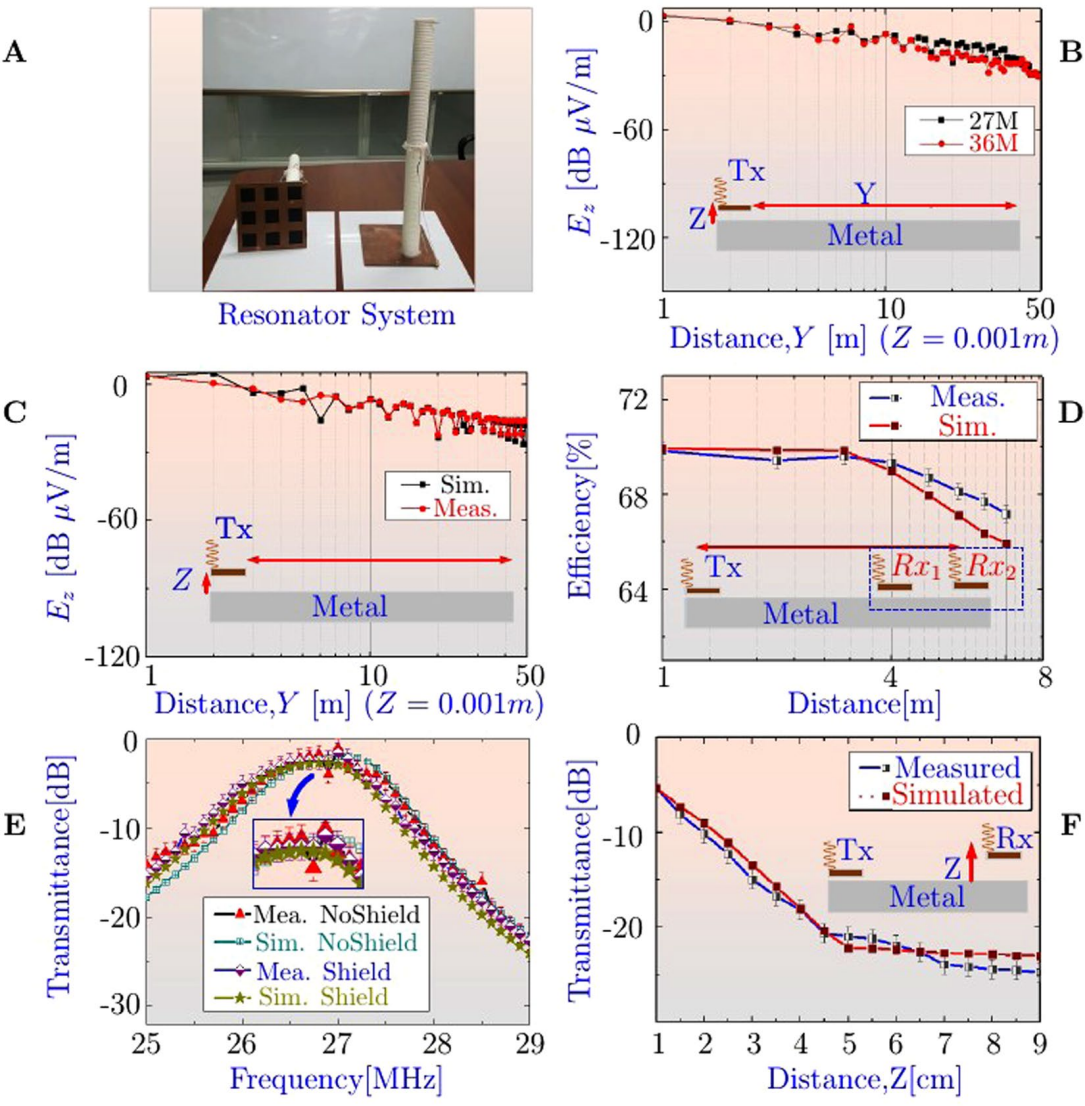
Experiment and Simulation results: Zenneck Wave at metal-air interface. (**A**) Ground Backed Impedance resonator system, with a half wavelength helical coil. (**B**) Experimental results of the Z component of the Electric field in the Y-direction 1 to 50 *m*, shows a slow attenuation rate. Two resonators with frequencies 27 and 36 *MHz* were designed and compared. The resonators were placed at a vertical distance of *Z* = 0.001 *m* above the metal surface. (**C**) Measured and simulated results comparison of E field attenuation along Y-direction at 27 *MHz*; *Z* = 0.001 *m*. (**D**) Multi receiver power transfer efficiency. (**E**) Experimental and simulated results of the transmittance parameters, when transmitting and receiving unit are under shield and no-shield conditions. (**F**) Evanescent field decay experiment.

The ZW properties of the proposed system have been experimentally observed and are presented in Fig. 3. The resonator system is shown in the Fig. 3A, dimensions and parameters are presented in the Supplementary Material (Fig. S9 and Table ST 2).

#### Frequency independent slow attenuation rate

The Fig. 3B, shows the comparison of the attenuation rate of the E-field[*dBμV*/*m*] for the two resonator systems designed for operating frequencies of 27 *MHz* and 36 *MHz*. The transmitter and receiver was fixed at a height of *Z* = 0.001 *m* above the metal surface. However, the receiver was moved along the interface (*Y*-direction) and the corresponding values were recorded^14^. It is observed that the E-field values along the metal show a slow attenuation rate, independent of the frequency. This property is consistent with the ZW’s as reported by Schelkunoff, Sarkar *et al*. and Barlow^14,15,18,20^. The Fig. 3C, shows the measured and simulated results of the attenuation rate at 27 *MHz*. The simulation was done using Ansys high-frequency structure simulator (HFSS). It is observed that the experiments and simulation model are in excellent agreement.

#### Multi receiver efficiency

The Fig. 3D, shows the multi receiver efficiency from 1 *m* to 8 *m* distance. Two receivers with identical loads were used of 20 watts each. The simulation result of the transmittance parameters are listed in the Supplementary Material (section 5). It is observed that the system efficiency varies between 66% to 62% for a range of 1 to 8 *m*. The power transfer metrics at 8 m and 15 m are listed in the Supplementary Material (Tables ST 4 and ST 5).

#### Leaky or partial metal shields

The Fig. 3E, shows the comparison of measured and simulated results of the transmittance parameters under leaky shielded and non-shielded conditions. The transmittance parameters were observed using the state-of-the-art vector network analyzer. The FEM model is in good agreement with the measured results. It is observed that the proposed system, unlike the coupled WPT systems, has the ability to perform without any significant efficiency degradation^2,23^. Detailed measurement results are further discussed in the *Power Transfer Metrics* section.

#### Evanescent field/exponential decay

An exponential E-field decay is also observed in the normal direction away from the metal-air interface (listed in Fig. 3F), consistent with the evanescent property of the ZW’s^10–12,14–16,18–21^.

#### FEM Simulation model and Sinking of Iso/Equi-phases

**Figure 4:**
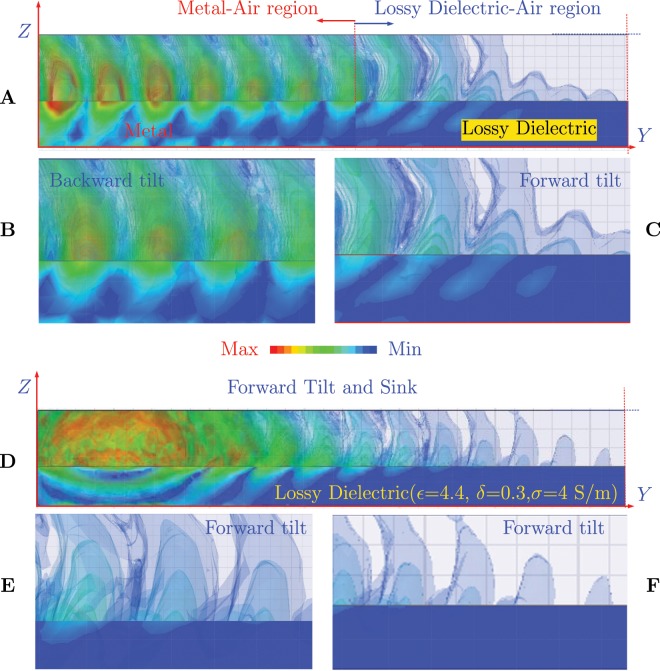
Simulation of Iso-Phases. (**A**) Half metal (Aluminum) and Half Lossy dielectric (*ε* = 4.4, *δ* = 0.3, *σ* = 4 S/m). (**B**) Inset View of Iso-Phases on metal. (**C**) Inset view of Iso-phases in lossy dielectric. (**D**) Iso-Phases for a complete lossy dielectric (*ε* = 4.4, *σ* = 0.3, *σ* = 4 S/m). (**E**) Inset view of Iso-Phases at the beginning of the lossy dielectric media. (**F**) Sinking of the Iso-phases into the lossy dielectric.

**Figure 5:**
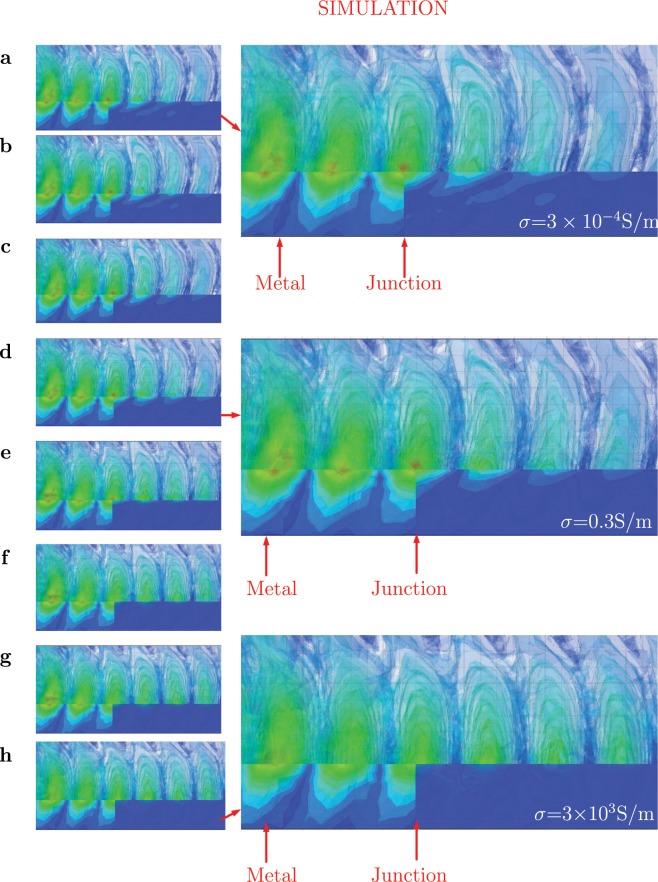
SIMULATION: Capture of the Iso-amplitudes and phases for different conductivities of the dielectric material in *S*/*m* (**a**) *σ* = 3 × 10^−4^. (**b**) *σ* = 3 × 10^−3^. (**c**) *σ* = 3 × 10^−2^. (**d**) *σ* = 3 × 10^−1^. (**e**) *σ* = 3 **(f**) *σ* = 30. (**g**) *σ* = 300. (**h**) *σ* = 3000.

The simulation of Iso-phases mentioned in the Fig. 2B, is shown in the Fig. 4A to D. The simulation set up; in the Fig. 4A two kind of materials are used comprising of equal lengths of metal and lossy dielectric. It is observed that the equi or Iso-phases are tilted backwards in the air, as long as metal-air interface exists as shown in Fig. 4A,B. Where as, for a case of Lossy dielectric-Air interface, the same Iso-Phases are tilted forwards in the air. When we look at the simulation of lossy dielectric-air interface as shown in Fig. 4D to F; the Iso-phases are titled forwards in the air, in the direction of propagation. The Fig. 4 and the Fig. 5A–H, confirms the implications of Eq. 1, i.e. the governance of the angle of tilt as per the media. This is one of the important facts brought out by the current study.

#### Eddy current effect

The current carried in the primary of the resonator coil, is effected by the eddy currents generated on the metal. This effect was reduced by increasing the spacing between the coil and the GBI resonator from 105 *mm* to 260 *mm* (Supplementary Material Fig. S3a).

#### Attenuation rate along different interfaces

If the proposed method is exciting Zenneck waves at the metal-air interface, then, they must also show similar properties across various other conductive media. The Fig. S3b, shows the attenuation characteristics across aluminium (conductivity, $\sigma =3.8\times {10}^{7}\,S/m$), iron ($\sigma =1.03\times {10}^{7}\,S/m$) and sea-water ($\sigma =4\,S/m$ and *ε* = 81). It is observed that the attenuation rate in seawater is faster than metal.

### Additional commentary: Non-Capacitive system

**Figure 6:**
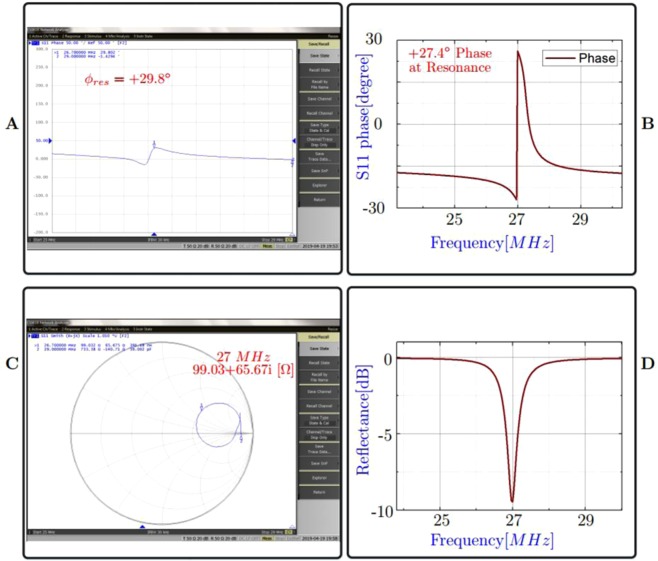
(**A**) Measured S11 Phase angle at resonance, having a value of 29.8°. (**B**) The HFSS simulation - S11 Phase angle at reosnance is positive, hence the ZW system is not a capacitive system. (**C**) Measured Smith Chart of the S11 shows a positive imaginary impedance quantity. Hence, inductive property is dominant. (**D**) HFSS simulation- Reflectance at 27 *MHz* resonance.

Recently, the Sommerfeld-Zenneck wave behaviour has been demonstrated in the centimeter range^30^. Interestingly, (the Fig. 2C top-view of^30^) of the article shows the simulation of the E-field modes identical to the Fig. S.15. At a first glance, the presented ZW system looks like a capacitive power transfer system. But, this is misleading, we need to look at the details of the phase of the reflectance parameters, when the transmitter is placed in the proximity of the metal. Figure 6A,B shows the measured and simulated phase angle at resonance to be +29.8° and +27.4°, respectively. Moreover, the Fig. 6C shows the smith chart results, which shows a positive value for the complex quantity of the impedance at resonance. Finally, Fig. 6D shows the reflectance parameters magnitude in *dB* having a resonance at 27 *MHz*. Ofcourse, all these observations are made by placing the transmitter on the metal, with an insulator. Based on the results of Fig. 6A to C, it can be concluded that the ZW system is *not a capacitive* power transfer system^31,32^. As per the available literature, the phase angle of the S-parameter and imaginary part of the impedance in smith chart must be **negative** for a capacitive power transfer system. Also, it is evidently clear that the Capacitive or coupled WPT systems **do not** exhibit propagating mode behavior.

### Power transfer metrics

**Figure 7:**
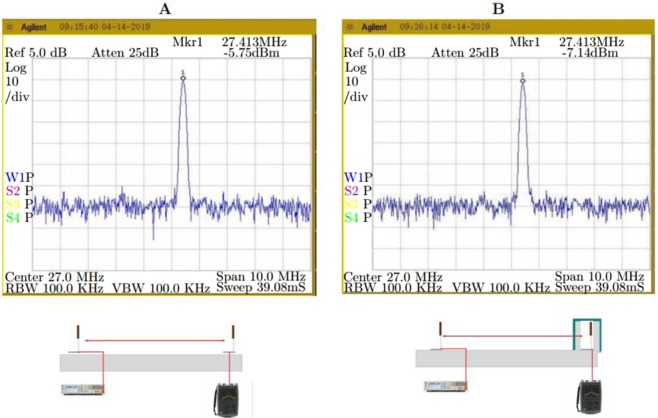
Measurement Spectrum analyzer under (**A**) open conditions (**B**) shielded conditions; distance between transmitter and receiver was at 15 *m*.

**Figure 8:**
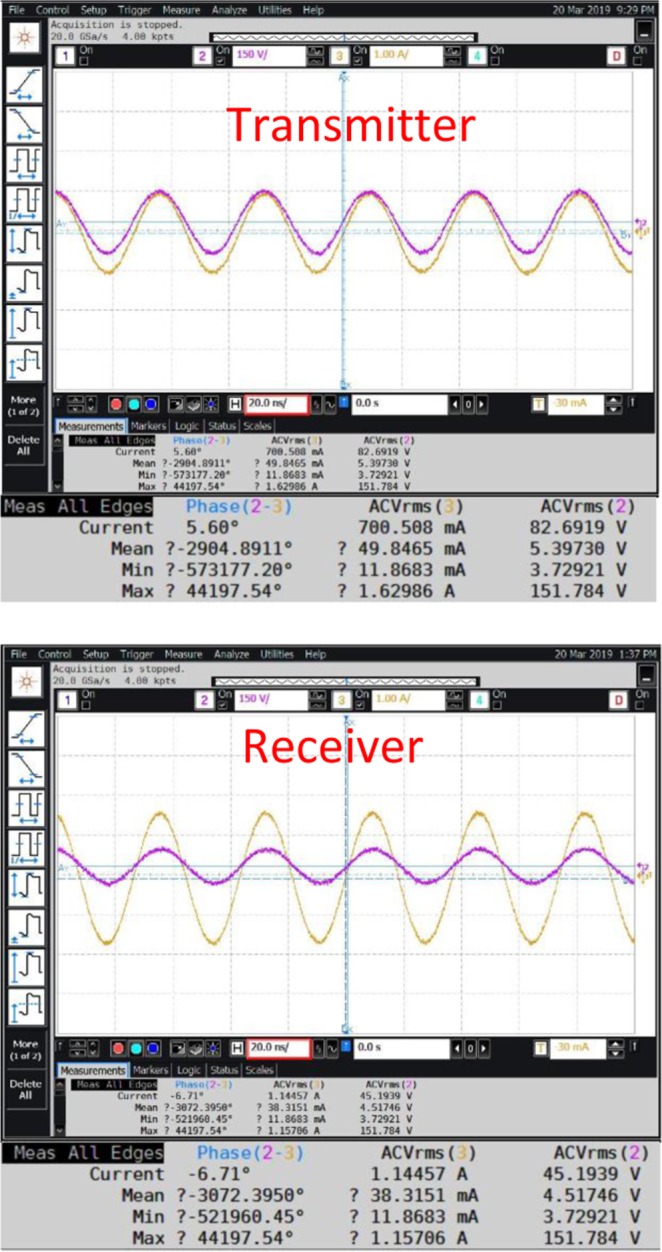
Measurement: AC RMS Current, Voltage and Phase across Transmitter terminals open-conditions; distance between transmitter and receiver was at 8 *m*.

**Figure 9:**
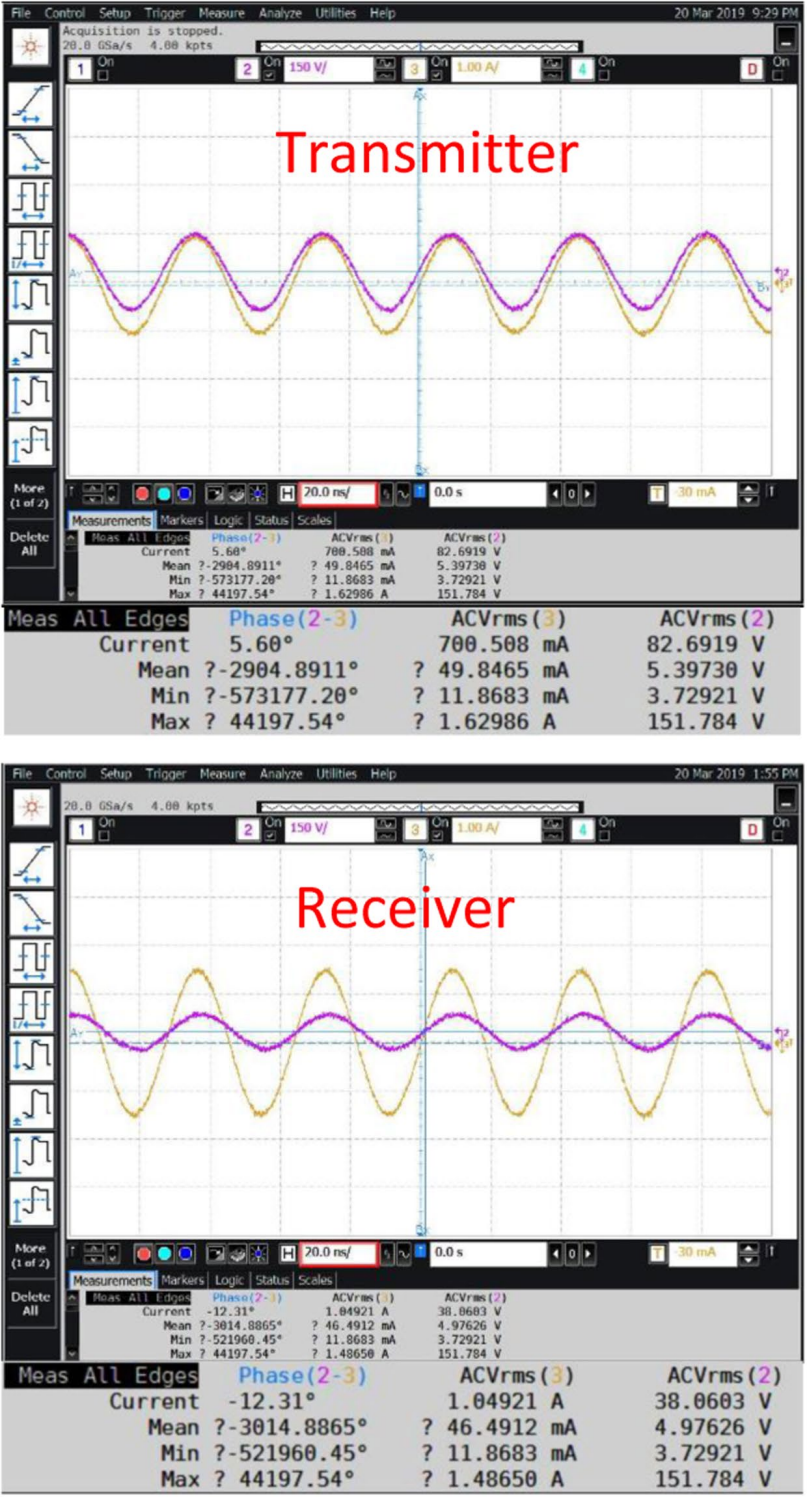
Measurement: AC RMS Current, Voltage and Phase across Transmitter terminals shielded-conditions; distance between transmitter and receiver was at 8 *m*.

The Fig. 7A shows the received absolute power under open conditions, the corresponding shielded conditions is shown in the Fig. 7B. Both these measurements are performed using spectrum analyzer (Agilent N9320B 9 *KHz*–3 *GHz*) on the receiver side. The transmitter is fed with 0 *dBm* from a signal generator (Agilent N5183A), the sweep conditions are start frequency 22 *MHz*, center frequency 27 *MHz* and the stop frequency is 32 *MHz*. The distance between transmitter and receiver is 15 *m*. The Fig. 8 shows the measured AC RMS current, voltage and phase values across transmitter and receiver (open conditions), transmission range is 8 meters. On the other hand receiver under shielded conditions is shown in the Fig. 9. A Keysight differential voltage probe N2790A 100 *MHz* and Keysight 1147B current probe was used for these measurements. An agilent mixed signal oscilloscope model MSO9254A was used for recording the measurements. Interestingly, the transmitter to receiver efficiency is high; $$\eta =[{V}_{R{x}_{RMS}}\times {I}_{R{x}_{RMS}}\times cos{\phi }_{Rx}/{V}_{T{x}_{RMS}}\times {I}_{T{x}_{RMS}}\times cos{\phi }_{Tx}]\times 100=[45.19\times 1.1445\times cos$$$$(-6.71)/82.85\times 0.7\times cos(5.68)]\times 100=89.1 \% $$. However, when one calculates the power amplifier to receiver efficiency, the figure drops to 64%. The input from signal generator is −4 dBm, the power amplifier [the model Prana DP 300] used has a gain of 53 dBm. Therefore, the total power fed into transmitter is 49 dBm, which is 79.4 Watts. This discrepancy can be explained by the existence of reflection losses at the transmitter end. Use of an appropriate impedance matching network on the load end can improve the overall efficiency, i.e. power amplifier to the receiver load. Under shielded conditions, the transmitter to receiver power transfer efficiency is 82.29%; the *power amplifier to receiver* efficiency is 57.19%. It is clear from these results, that the proposed ZW system has a clear advantage when it comes to environments where leaky metal shields are present, such as - *industrial pipelines, aerospace, wireless solutions for space payloads, wireless sensors for electric vehicles, marine vessels, smart shipping containers, modular metal buildings etc*.

## Discussions

We have demonstrated the excitation of waves on metal surfaces that can be used for delivering electrical power to multiple devices. The waves show slow attenuation property similar to ZW’s along the metal-air interface and can be used to deliver efficient power to devices upto 8 meters using the current design. We also show that ZW’s can be used for transmitting power across partial metal enclosures. Therefore, the resonator system has the ability to overcome electromagnetic shielding and can be used for delivering power to devices under leaky metal enclosures. Thus, agreeing with the hypothesis that excited waves are non-radiative in nature (ZW, SP, SW are non-radiative). Power transmission to multiple receiving resonators with uniform efficiency has also been established experimentally and shows excellent agreement with simulation. The simulation result is compared with coupled mode power transfer system in the Supplementary (Fig. S8). Existing *coupled mode* WPT systems undergo peak splitting when multiple receiving units are involved. Our study shows that using a wave-based mode of transmission, we can solve this issue. The efficiency of power transmission increases when multiple receiving units are present, as the power is uniformly spread across the metal surface. This kind of increase, due to multiple receiving units was also observed in a widely followed article, where weakly coupled WPT system is used^33^. The maximum value of E and H-field emitted by the system is 34% and 89% lower than the permitted values, regulated by the ICNIRP guidelines at this frequency (Supplementary material Fig. S10, S11 and Table ST 3). Thus, this system should not pose “occupational hazard” to human operators. The proposed system has no effect on other devices operating in vicinity (Supplementary Material demo video links)^34^.

### Advantages and limitations

Since ZW, SW, SP and SPP have an evanescent field, the transceivers need to be in proximity to the interface, on the other hand free space wave bases systems do not have this limitation. However, most free-space wave systems have limitations in power handling, efficiency and can not perform in the presence of leaky shielded environments. We have shown that the proposed ZW in this report has handled 65 *watts*. In its present form the presented system can handle upto 700 *Watts* of power. Beyond 1 *kWh*, the dielectric material and insulator material has to be replaced by air or ceramic in order to avoid dielectric breaking.

## Supplementary information

Supplementary Information.
