# Supplementary material for: Experimental Realization of Zenneck Type Wave-based Non-Radiative, Non-Coupled Wireless Power Transmission
Source: Sci Rep. 2020 Jan 22;10:925. doi: 10.1038/s41598-020-57554-1 (PMC6976601; doi:10.1038/s41598-020-57554-1)
Supplement: Supplementary file 1 — Supplementary Information. [file 41598_2020_57554_MOESM1_ESM.pdf]

Supplementary Material for

Experimental Realization of Zenneck Type Wave-based Non-Radiative, Non-Coupled Wireless Power Transmission

Sai Kiran Oruganti,[1+,2*] Feifei Liu,[2] Dipra Paul,[1] Jun Liu,[3+]
Jagannath Malik,[1] Ke Feng,[2] Haksun Kim,[1] Yuming Liang,[2]
Thomas Thundat [3+] and Franklin Bien.[1*+]

December 2, 2019

1 Experimental setup

The Fig. S 1 shows the experimental setup used in this study.

2 Electrical Length: Half Wave Helical Transformer

Throughout 2015-18, authors conducted the experiments to study the voltage oscillation across the GBI resonator system. One such experiment carrying out power transfer across 80 mm metal wall can be seen in fig. S 2. This experiment uses the ground plate and wire arrangement to sustain a meaningful voltage oscillation across the terminals of the load (40 watts halogen).

Based on the experimental findings shown in fig. S 2, the authors tried to replace the equivalent RLC lumped elements(unsuccessfully so). The reasons behind the failure of RLC lumped elements based counterpoise were carefully and qualitatively investigated, through a series of experiments. It was observed that the planar structure of the receiver was unable to sustain a significant value of voltage and current. The gap between the mesh and ground layer is g=1.5 mm. At a target frequency of $27MHz$, $g << \lambda/4$. The largest dimension of the transceiver system is $A_x = 150\ mm$, which is $<< \lambda/2\pi = 1767mm$ at target frequency of $27MHz$. Thus, the proposed receiver in its present form is electrically

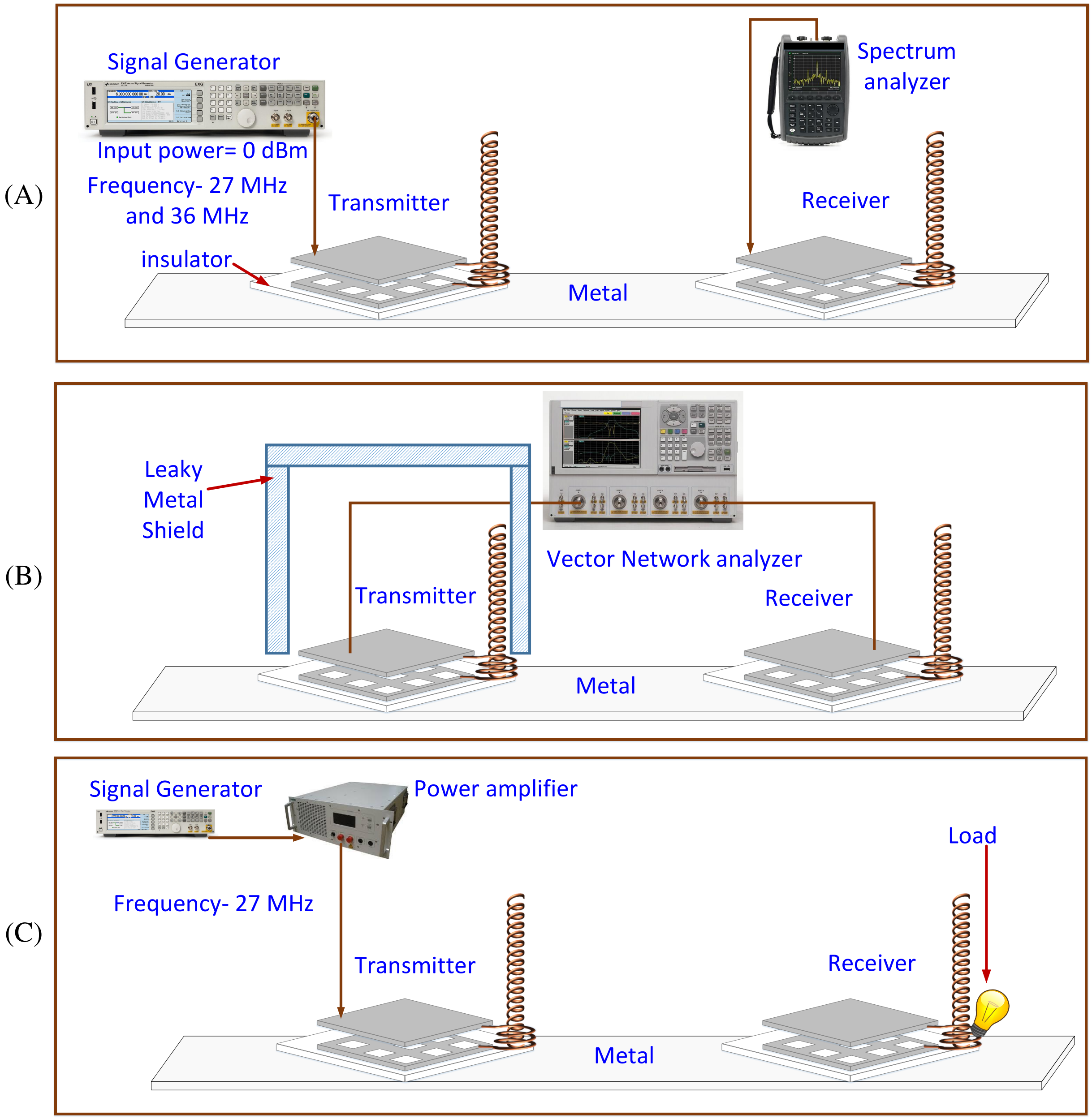

Fig. S 1 – Experimental setup (A) E-field measurement (B)Transmittance parameters (C) Power transfer

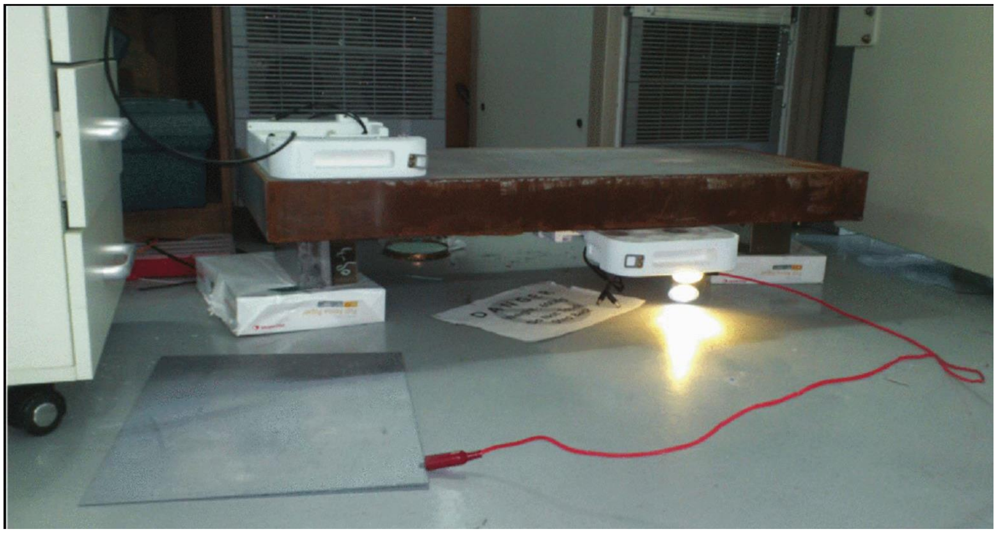

Fig. S 2 – Earlier experiments: Power transfer across 80 mm using the ground-wire counterpoise on the receiving unit to sustain voltage oscillation

small [1, 2].
It has been observed that the dimensions of the lumped RLC elements become the part of the over all electrical length of the antenna beyond $900MHz$ [3, 4]. However, in the HF regime, the dimensions of the RLC elements can not provide the appropriate electrical length. Electrically small antenna's have poor radiation efficiency, which in turn also hinders the receiving capabilities of high frequency antennas [1, 2]. However, the radiation efficiency of the receiving antennas can be improved.

As per the standard definition the GBI structure is an electrically small antenna $ka << 1$[1, 2]. Based on the analysis presented in [2] the quarter wavelength counterpoise is like a mono pole. One such reading of current and voltage values along the length of the quarter wavelength wire counterpoise is listed in table 1.

Table ST 1 – Current and voltage across the counterpoise

Length	AC RMS Current (mA)	AC RMS Voltage (V)
$\frac{1}{3}(\lambda/4)$	360.74	13.0515
$\frac{2}{3}(\lambda/4)$	362.5	22.19
Full Length ($\lambda/4$)	361.1	43.82
Tx side	1200	56
Rx terminals	796	12

However, this is not a practical dimension for the purpose of power transmission. A multi turn helical alternative would be a better choice. The radiation resistance in this case is related to the area, A_{loop} of a helical loop of N turns by [1, 2]:

$$R_{rh} = 320\pi^2 N^2 \left(\frac{A_{loop}}{\lambda^2} \right)^2 \quad \text{(S1)}$$

Attaching a multi-turn helical electrical conductor to one of the copper elements of the GBI resonator would simply change the resonance conditions[5, 6]. In order to prevent the change of resonant conditions and to drive the reference voltage of the GBI structure to a high level, the half wave helical coil was incorporated in the tesla transformer fashion.

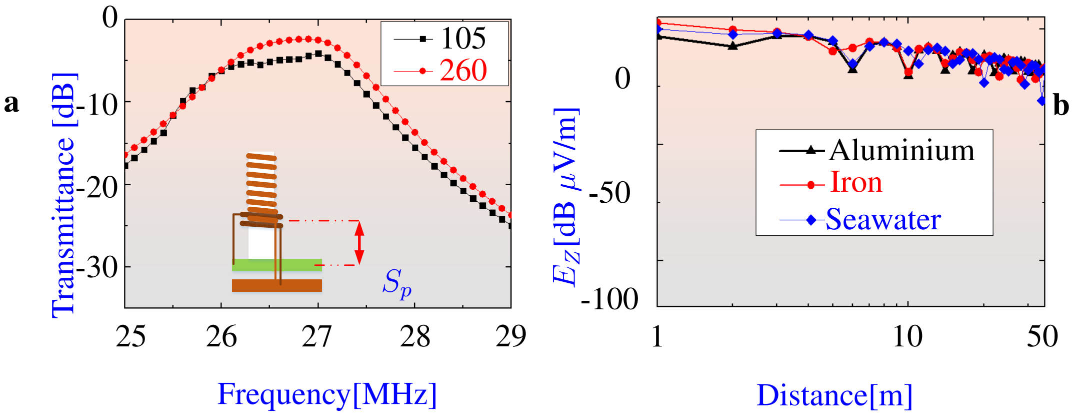

Fig. S 3 – FEM Simulation Model. (a) Representative diagram of the mode profiles, completing in free space. The Zenneck wave sinks into the lossy dielectric. (b) Transmittance parameters when the coil is placed at a vertical spacer of S_p =105 mm and 260 mm from the ground layer of the resonator. (F) Attenuation of E-field in the transverse direction along the interface of Air-Aluminium, Air-Iron, Air-Seawater.

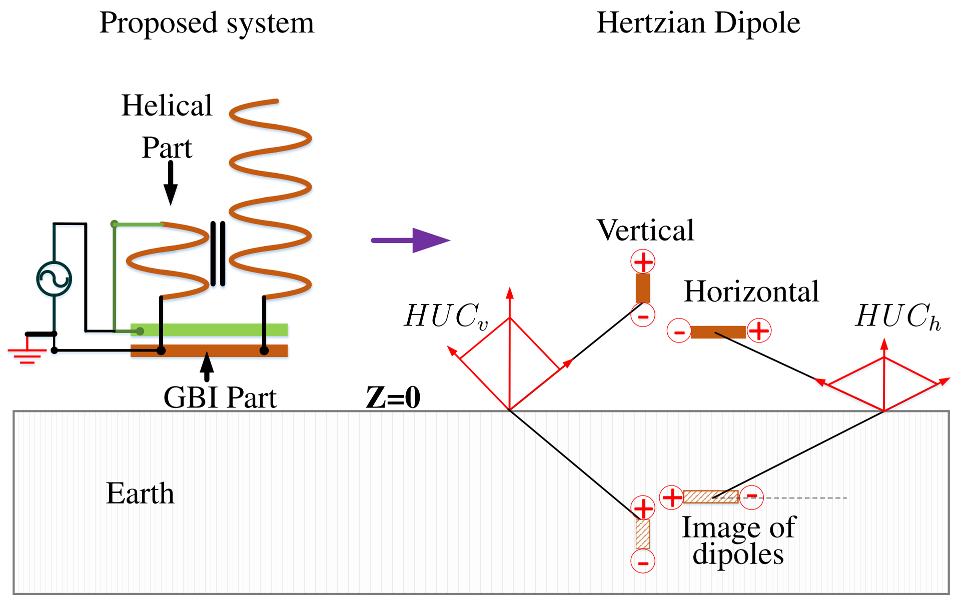

Fig. S 4 – Reflection by a conductive earth surface: Sommerfeld's formalism

3 Sommerfeld's Analytical Formalism: Vertical and Horizontal Hertz dipole over metal-planes

Let us first review Sommerfeld's analytical formalism. Retaining the notation as suggested by Sommerfeld [14]; let ω be the frequency in radians, $k = 2\pi/\lambda = \omega/c$ be the wavenumber and c be the speed of light. Their corresponding relation with the Hertzian potential:

$$\prod = \frac{1}{r} e^{i(kr-\omega t)} \tag{S2}$$

From the electrodynamics point of view, Hertzian potential is a vector and now on shall be denoted by $\overrightarrow{\prod}$. The proposed system is electrically small and hence, the current carried by the antenna has no phase variations on the primary side of the helical coil and the GBI structure. Please note, there will be no current carried by the secondary helical coil, as it is in principle an open-circuit. As shown in Fig. S 4, the interface between metal and free space exists as $Z = 0$. The tangential E field component would be zero in accordance with Maxwell's equation [8]:

$$(k^2.\overrightarrow{\prod} + grad\ div\overrightarrow{\prod})_{tang} = 0 \tag{S3}$$

This is satisfied by combined effect of the dipoles with their corresponding mirror images formed in the metal as shown in the Fig. S4. Sarkar et al. also used Schelkunoff integrals for images formed in imperfect earth[8, 9]. Evidently, there are two kinds of dipoles which need to be considered in the case at hand.

1. Vertical dipole, originating from the primary coil at a distance h above $z = 0$. Leading to expression for the vector potential:

$$\prod = \prod_z = \frac{e^{ikR}}{R} + \frac{e^{ikR'}}{R'} \begin{cases} R^2 = r^2 + (z-h)^2, \\ R'^2 = r^2 + (z+h)^2. \end{cases} \tag{S4}$$

   In the Fig. S4, the term HUC_v denotes the hypothetical unit charge at $Z = 0$, thus resulting to a force in the Z direction. The $E_{tang} = 0$.

2. The Horizontal dipole arising from the GBI structure can be expressed by:

$$\prod = \prod_x = \frac{e^{ikR}}{R} - \frac{e^{ikR'}}{R'} \tag{S5}$$

   The horizontal dipole forms a hypothetical unit charge, denoted by HUC_h, resulting into a force in the Z direction and thus causing the $E_{tang} = 0$.

3. If one applies a limit $h \to 0$, then the vertical dipole results in a $\prod = 2.e^{ikR}/R$, while horizontal dipole's vector potential vanishes. In the present case, we have placed the resonator system at a distance of $h = 0.001mm$. Its, contribution to the directional characteristic is considered for the final evaluation.

4. Therefore, the generalized forms of the equations S4 and S5, with amplitudes A and B can be expressed as:

$$\prod = \prod_z = A.\frac{e^{ikR}}{R} \tag{S6}$$

   The horizontal dipole can be expressed as a quadrupole as:

$$\prod = \prod_z = B.\frac{\partial}{\partial x}\frac{e^{ikR}}{R} \tag{S7}$$

The complex refractive index n for the metal is related as:

$$n = \sqrt{\frac{1}{\epsilon_0}(\epsilon + i\frac{\sigma}{\omega})} \tag{S8}$$

where σ is the conductivity and ϵ_0 and ϵ are the free-space permittivity and relative permittivity, respectively. The wavenumber in the free-space (k) and the metal (k_M) can be related as:

$$k_M = nk \tag{S9}$$

3.1 Three regions in the interface

The height of the location of the Hertzian dipole is denoted by h as mentioned earlier. There are three distinct regions to be considered for the analysis.

3.1.1 Region-I: $z > h$ (Conducting Earth-Air interface)

In the analysis by Sommerfeld on the problem of dipole over arbitrary ground with finite conductivity, it was assumed that in addition to a primary stimulus originating from the antenna at $r = 0$ and $Z = h$, there exists a secondary stimulus, due to localized charge oscillations in the earth. Dealing with the problem in the cylindrical polar coordinates (r, ϕ, z), we shall use the eigenfunctions u and eigen values Λ.

$$\begin{aligned} \prod_{prim} &= \int_0^\infty j_0(\Lambda r) e^{-M(z-h)} \frac{\Lambda d\Lambda}{M} \\ \prod_{sec} &= \int_0^\infty F(\Lambda) j_0(\Lambda r) e^{-M(z-h)} d\Lambda \end{aligned} \tag{S10}$$

Where, $F(\Lambda)$ is the undetermined spectral distributions in the Λ-continuum of the eigenfunctions. The quantity, j_0 is the Bessel's function. The quantities $u = j_0(\Lambda r)\cos Mz$ and $k^2 = \Lambda^2 + M^2$.

3.1.2 Region-II: $h > z > 0$ (Air)

The primary and secondary stimulus exists in this case as well, represented by the analytical relations similar to S10:

$$\begin{aligned} \prod_{prim} &= \int_0^\infty j_0(\Lambda r) e^{+M(z-h)} \frac{\Lambda d\Lambda}{M} \\ \prod_{sec} &= \int_0^\infty F(\Lambda) j_0(\Lambda r) e^{-M(z+h)} d\Lambda \end{aligned} \tag{S11}$$

The equations S10 and S11 follow continuity behaviour of the $\prod$ field at the boundary for an arbitrary $F(\Lambda)$.

3.1.3 Region-III: $0 > z > -\infty$ (Earth)

There would be no primary stimulation in this case; the field denoted by $\prod_M$ must be continuous throughout.

$$\prod_M = \int_0^\infty F_M(\lambda) j_0(\Lambda r) e^{+M_M z - Mh} d\Lambda, \qquad M_M^2 = \Lambda^2 - k_M^2. \tag{S12}$$

The equations S11 and S12 satisfy the Maxwells boundary conditions of continuity, their resulting relation would be(region II and region III):

$$\int_0^\infty j_0(\Lambda r) e^{+M(z-h)} \frac{\Lambda d\Lambda}{M} + \int_0^\infty F(\Lambda) j_0(\Lambda r) e^{-M(z+h)} d\Lambda = n^2. \int_0^\infty F_M(\lambda) j_0(\Lambda r) e^{+M_M z - Mh} d\Lambda \tag{S13}$$

The first term on the LHS of the equation S13 is the $\prod_{prim}$ and the second term is $\prod_{sec}$ from the equation S11. The RHS is the S12 multiplied by n^2 which comes from equation S8. Solving the above equations in the complex plane as shown in Fig. 5, results in the four Riemann sheets($\Lambda = k$ and $\Lambda = k_M$) and simplifying in their bessel function form, we get the Hankel function of type 1 and 2:

$$j_0 = \frac{1}{2}(H_0^1 + H_0^2) \tag{S14}$$

Substituting $\varrho = \Lambda r$ the equation S14 becomes:

$$j_0 = \frac{1}{2}(H_0^1(\Lambda r) + H_0^1(\Lambda e^{i\pi} r)) \tag{S15}$$

Notice, only the Hankel function of the first type remains. Integration through the path w_1 and w_2, we obtain the following integral:

$$\int_{W_1} j_0(\Lambda r) f_{arb}(\Lambda^2) \Lambda' d\Lambda' = \frac{1}{2} \int_W H_0^1(\Lambda r) f_{arb}(\Lambda^2) \Lambda d\Lambda \tag{S16}$$

Where, $f_{arb}(\Lambda^2)$ is an arbitrary function of Λ^2. Thus, the real integral has been converted into a complex integral closing at infinity. Thus the solution for the primary stimulation:

$$\prod_{prim} = \frac{1}{2} \int_w H_0^1(\Lambda r) e^{-M|z|} \frac{\Lambda d\Lambda}{M} \tag{S17}$$

$$\prod = \int_w H_0^1(\Lambda r) e^{-Mz} \frac{n^2 \Lambda d\Lambda}{n^2 M + M_M} \tag{S18}$$

It is evident from the fig. 5,in the positive imaginary half plane, the Hankel function of the first order $H^1(\Lambda r)$ vanishes at infinity. The path of integration avoids the loops Q

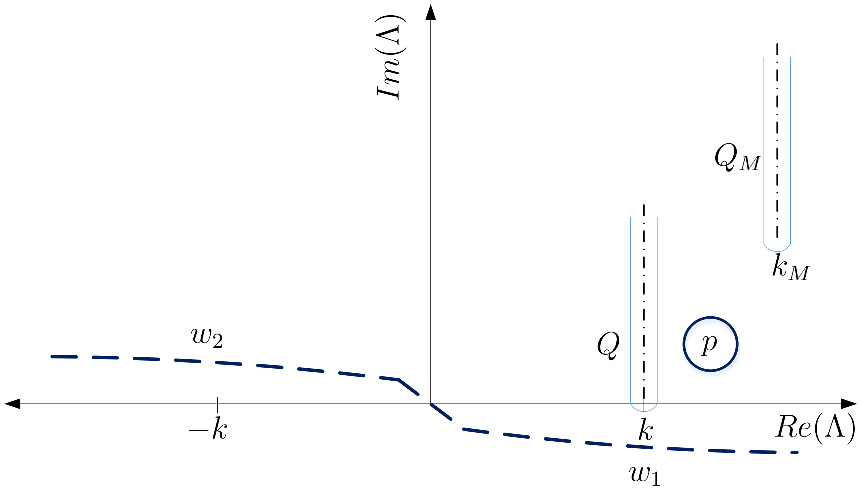

Fig. S 5 – Integration paths w_1 and $w = w_1 + w_2$, the path deforms around the branch-cuts Q, Q_M and the pole p. This was published in Sommerfeld's work.

and Q_M. The singularity exists for integrand as shown in equation S18, the denominator $n^2M + M_M$ would vanish at:

$$\Lambda = p$$

We have three components Q,Q_M and p, the contribution of Q_M can be safely ignore for the large values of $|k_M|$ because the Hankel function decays exponentially at large distances from the real axis. By applying method of residues and setting up the relationship $\widetilde{K} = k_M^2/(\sqrt{p^2 - k^2}) + k^2/(\sqrt{p^2 - k_M^2})$ one arrives at the following new relations for the vector potential:

$$\prod = 2\sqrt{2\pi i/pr}\frac{k_M^2}{\widetilde{K}}e^{ipr-\sqrt{p^2-k^2}z}, \qquad z \geq 0, \tag{S19}$$

$$\prod\nolimits_M = 2\sqrt{2\pi i/pr}\frac{k^2}{\widetilde{K}}e^{ipr+\sqrt{p^2-k_M^2}z}, \qquad z \leq 0, \tag{S20}$$

Finally the above equations can be written in a modified form for a wave:

$$\prod = A.k_M^2 e^{ipx-\sqrt{p^2-k^2}z}, \tag{S21}$$

$$\prod\nolimits_M = A.k^2 e^{ipx+\sqrt{p^2-k_M^2}z} \tag{S22}$$

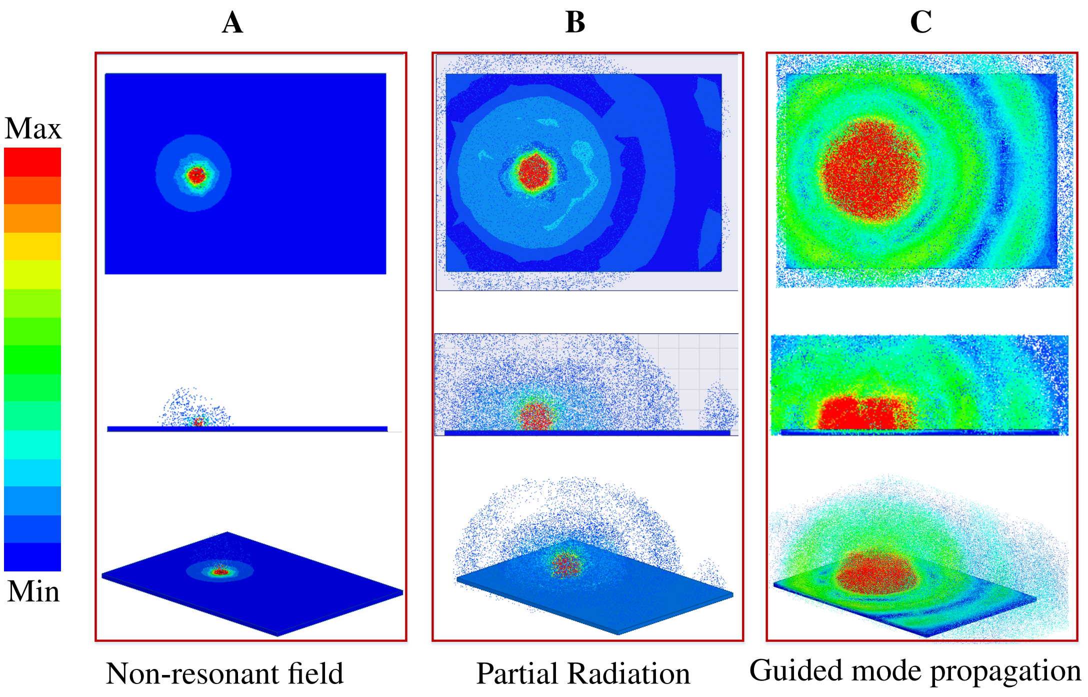

Fig. S 6 – Superposition of fields: (A) GBI resonator a case of horizontal dipole (B) Half-wave Helical coils as a case of vertical dipole (C) Proposed system, a combination of GBI and Half-wave Helical

Where, A is a slowly varying amplitude factor, the equation S21 and S22 describe the Zenneck waves, which were originally proposed by Jonathan Zenneck in 1907.

3.1.4 Controversy

There are several controversies pertaining to the above analysis. Especially, the placement of the pole "p". Evident from Jangal et al. **"...the location of the pole "p" with respect to the integration path determines the kind of the excited Surface Wave."** [13]

$$P = -\tilde{Z}_s.\omega.\epsilon_0 \tag{S23}$$

Where, $\tilde{Z}_s$ is the complex surface impedance of the dielectric. Furthermore, the following equation obtained by Jangal et al. points to the center-stage of exact controversy[13]:

$$H_y(x,z)|_{z=0} = -i.k_z.e^{i.\sqrt{k_0^2-k_z^2 x}} + \frac{1}{\pi}\int_0^{\infty} \frac{p^2 e^{i.\sqrt{k_0^2-k_z^2 x}}}{p^2-k_z^2}.dp \tag{S24}$$

Take a note that the first term in the equation S24 is the ZW term (negative term), whereas the second term is the an equally contributing term with a positive term [13].

3.1.5 The Issues with Sommerfeld's Analysis

Perhaps the core of the controversy lies in the way the poles have been placed and the way the integral path has been deformed in fig S5. Sarkar et al. suggest the pole placement in Sommerfeld's analysis as shown in fig S7.

3.2 Analysis for Metals

Re-writing the Original Sommerfeld integral in the form suggested in **??**, the Hertzian integral for reflected wave from the homogeneous media from the filled half space. For convenience, let us also rewrite k and k_M as k_1 and k_2:

$$\prod_{1z}^{ref} = \int_0^{\infty} R(\Lambda) J_0(\Lambda_{rho}) e^{-(z+h)\sqrt{\Lambda^2-k_1^2}} \frac{\Lambda}{\Lambda^2-k_1^2} d\Lambda \tag{S25}$$

Here, $R(\Lambda)$ is the coefficient of reflection, also represented by $\Gamma(\lambda)$ in several published text and is defined as:

$$R(\Lambda) = \frac{\epsilon\sqrt{\Lambda^2-k_1^2} - \sqrt{\Lambda^2-k_2^2}}{\epsilon\sqrt{\Lambda^2-k_1^2} + \sqrt{\Lambda^2-k_2^2}} \tag{S26}$$

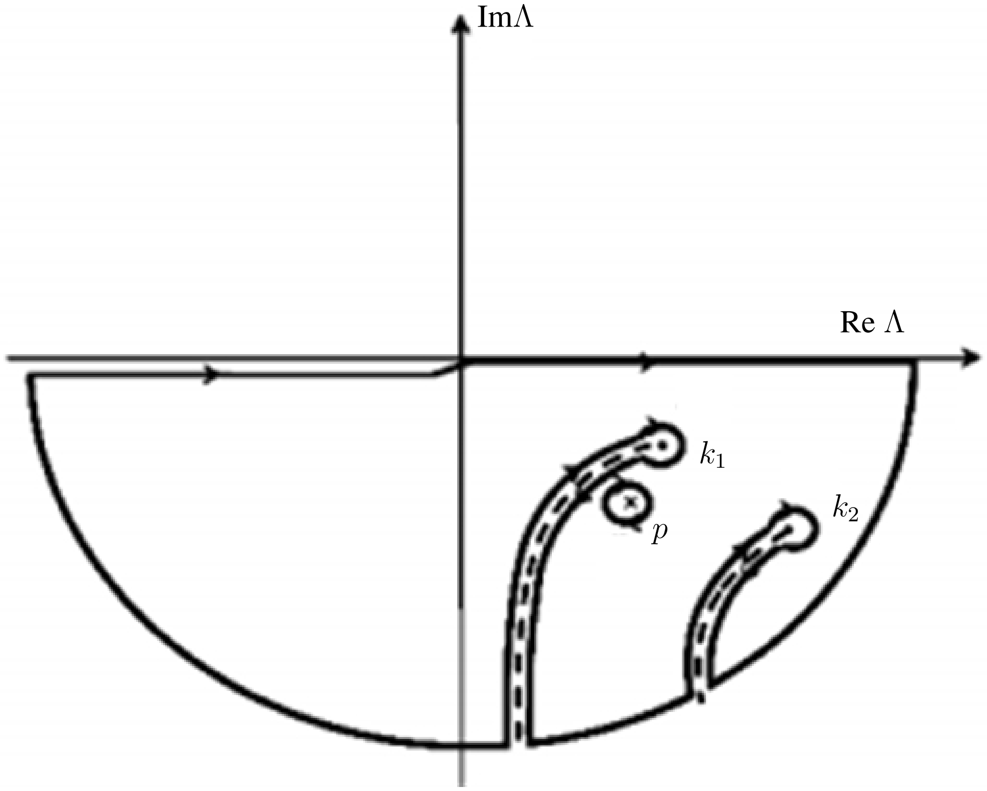

Fig. S 7 – The actual position of poles in the lower complex plane. Note, K is represented by k_1 and K_M by k_2

The roots to the equation S26 would give rise to 4 Riemann sheets in the complex plane, namely

$$\begin{array}{l} Sheet1 : Real(\sqrt{\Lambda^2 - k_1^2}) > 0 \quad and\ Real(\sqrt{\Lambda^2 - k_2^2}) > 0 \\ Sheet2 : Real(\sqrt{\Lambda^2 - k_1^2}) < 0 \quad and\ Real(\sqrt{\Lambda^2 - k_2^2}) > 0 \\ Sheet3 : Real(\sqrt{\Lambda^2 - k_1^2}) > 0 \quad and\ Real(\sqrt{\Lambda^2 - k_2^2}) < 0 \\ Sheet4 : Real(\sqrt{\Lambda^2 - k_1^2}) < 0 \quad and\ Real(\sqrt{\Lambda^2 - k_2^2}) < 0 \end{array} \tag{S27}$$

As per equation S27; only permissible Riemann sheet is the sheet 1. On this sheet the numerator of equation S26 or the zeros are known as the Brewster zeros.

The permittivity of metal be represented as a complex quantity of the form: $\epsilon = \epsilon^{'} - j\epsilon^{''}$; as per [15].

- For Lossy Earth: When $\epsilon^{''} >> |\epsilon^{'}|$ the pole is located either left or right of k_1 in fig. 7. More specifically when $\epsilon^{'} > -3/4$ the pole is on the left and when $\epsilon^{'} < -3/4$ the pole is on the right.
- Likewise, for Dry ground at high frequencies and Noble metals in visibile light: $\epsilon^{''} << |\epsilon^{'}|$. The pole is located on the left of k_1 for $\epsilon^{'} > 3/4$ and to the right for $\epsilon^{'} < 3/4$.

Based on the above, the metal below plasma frequencies exhibit negative for the real part of the ϵ, while the imaginary part is high value as per the Drude's model. Therefore the condition of $\epsilon^{''} >> |\epsilon^{'}|$, is readily satisfied. Also, $\epsilon^{'} > -3/4$, hence the pole resides on the left of k_1. This falls under the classification of high loss ZW.

4 Multi-receiver characteristics Comparison with coupled systems

The Fig. S 8 A and B show the well noted frequency splitting phenomena in case of coupled WPT systems. Whereas, the Fig. S 8 C and D, show the case of the proposed Zenneck Wave based WPT system, the power is uniformly received across both the receivers.

5 ICNIRP field compliance

One of the critical questions is the occupational hazard exposure from the proposed system, if used on board marine vessels, smart shipping containers and home IOT device

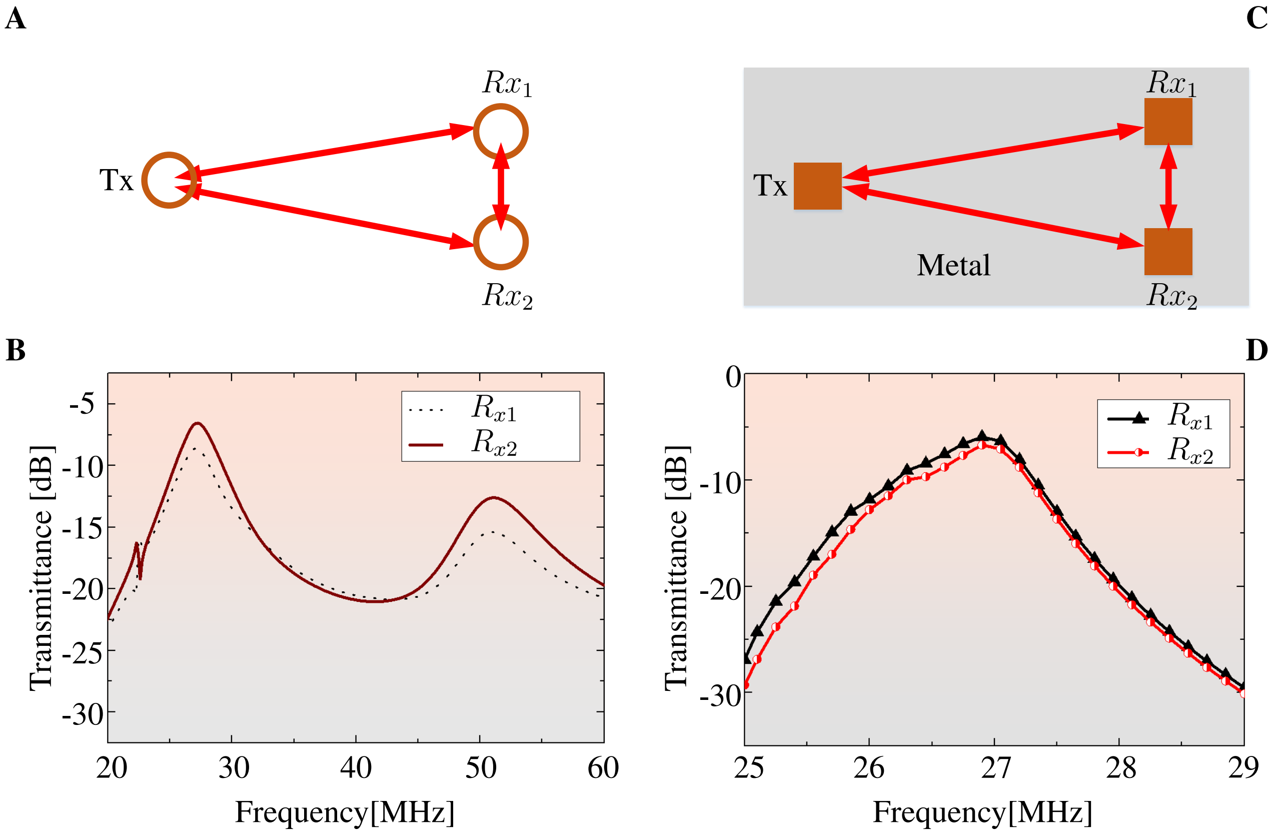

Fig. S 8 – Simulated (A) Inductively coupled WPT system. (B) Inductively coupled Transmittance parameters for multiple receivers. Frequency splitting is observed, when both the receivers are in proximity. (C) Zenneck Wave WPT system. (D) Zenneck Wave Transmittance parameters for multiple receivers. No frequency split and uniform power transfer is observed.

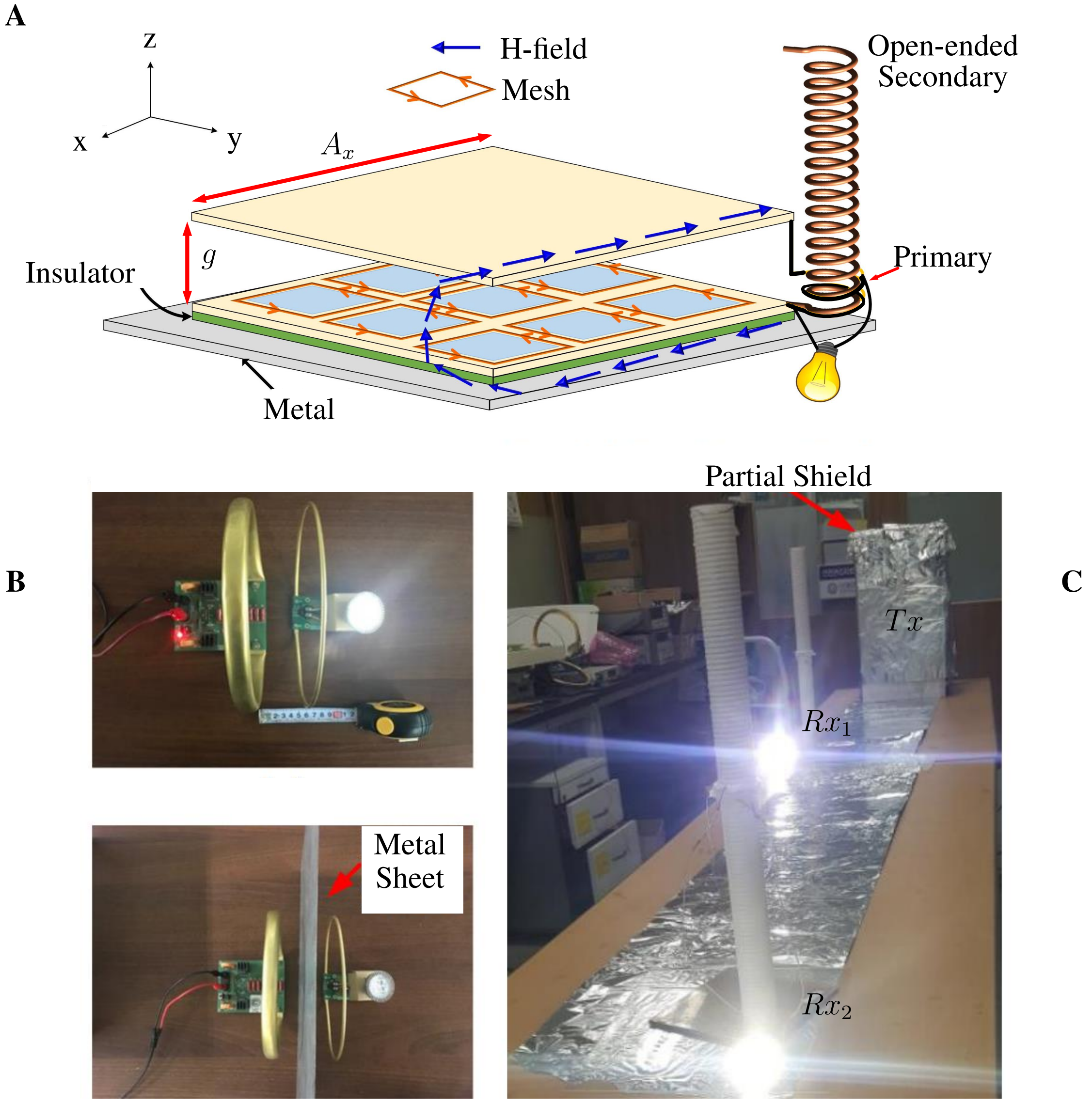

Fig. S 9 – Schematics and Demonstration (A) The receiver schematic diagram with load connected across the GBI structure's terminals $A_x = 150mm$,$g = 1.5mm$, $mesh\ slot = 30mm \times 30mm$; (expanded view of GBI) (B) EM shielding: Inductively coupled power transfer system under a plane metal sheet (C) Proposed Zenneck wave system, transmitter housed inside a partial shield illuminating multiple receivers Rx_1 and Rx_2

Table ST 2 – Coil details for Transmitter and Receiver

Parameter	Specifics
Secondary turns	52
Radius Secondary	18 mm
Primary Turns	2
Radius Primary	20 mm
Coil pitch in primary and secondary	4.3 mm
Coil to Rx ground spacer	230 mm
Secondary Coil Inductance	13.2 μH
Secondary Coil self capacitance	3.315 pF
Primary Coil Inductance	0.104 μH
Primary Coil self capacitance	1.208 pF

charging. Therefore, it was necessary to test the international commission for non ionizing radiation protocol compliance of the proposed system. The state-of-the-art Narda field measurement analyzer was used to record the E and H-field values across the spectrum of $1MHz$ to $30MHz$. The power fed into the transmitter was 65 $watts$. The fig.S 10 shows one of the several chosen position of the probe, which recorded maximum field intensities at the receiver and the edge of the metal. Fig.S 11, shows the recorded values of the Narda field test analyzer measurements. As per the ICNIRP regulation, between 10 MHz and 400 MHz the maximum permissible value of E -field is 61 V/m [21]. The maximum permissible regulatory value for H-field 0.16 A/m. The measured values of E and H-field in the proposed system is 40.3 V/m and 0.018 A/m, respectively, at $13.3MHz$. Implying, that the proposed system generates a maximum E-field of 33.9 $\%$ and H-field of 88.7 $\%$ lower than the regulation. Hence, the proposed system is safe for operation for human operators [21].

Table ST 3 lists the recorded field intensity values at various positions. The highest field values are recorded at the position 1 as shown in fig.S 10. The lowest is observed at position 3. A slightly higher value as compared to position 2 and 3 is observed at position 4. This slight rise in value can be attributed to the radiating power at the sharp corners of the metal. Its noteworthy, that the input power into the transmitter was kept at a higher value (65 $watts$) than the intended power value of 50 $watts$ for household usage. When

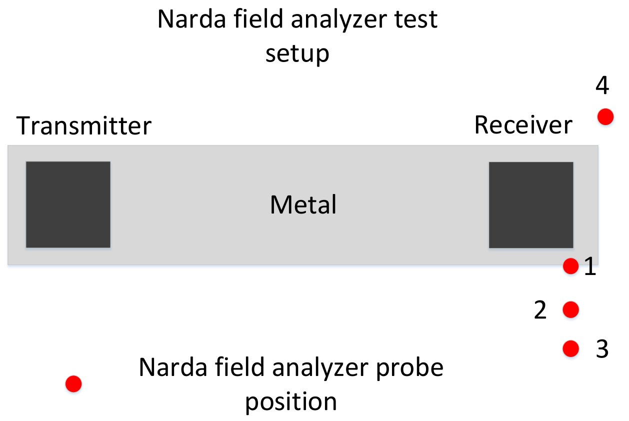

Fig. S 10 – Measurement setup for ICNIRP field compliance test. The red circles show the positions of the isometric probe of the field analyzer for measuring the field intensities.

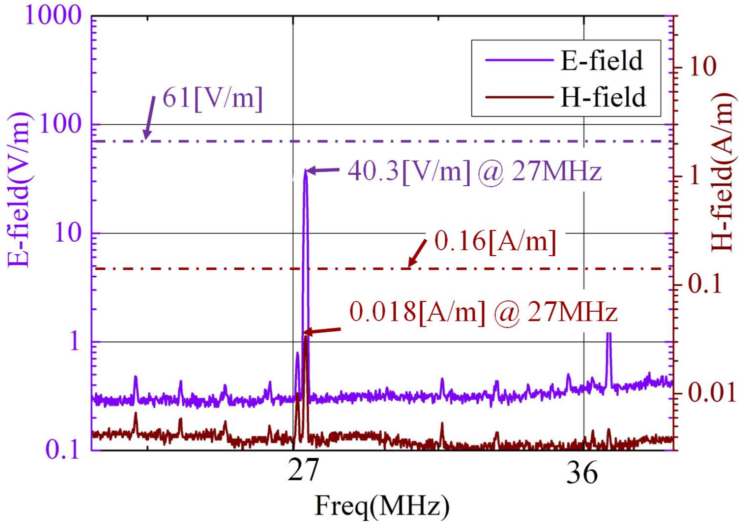

Fig. S 11 – Recorded EM fields for position 1 using a Narda STS EHP-200A Electric and Magnetic Field Analyzer; the solid lines represent the measured values, the dashed lines the regulatory reference(61V/m and 0.16 A/m)

Table ST 3 – Recorded E and H field results

Position	E $[V/m]$	H $[A/m]$	Details
1	40.3	0.018	2 mm vertical distance from metal
2	17.09	0.06803	100 mm horizontal
3	11.04	0.0205	150 mm horizontal 150 mm vertical from metal
4	19.7	0.073	100 mm corner from metal

two receivers are used, the value of E and H fields drop significantly below the public exposure regulations [21].

5.1 Multiple Receiver Capability

Power was transmitted along a 8 m metal sheet, the receivers were placed in various arbitrary configurations. The power reception was uniform despite of the configuration. However, beyond 8 m, a marginal degradation in power reception of the farthest receiver with respect to transmitter was observed. The table ST 4 lists the power transfer metrics for multiple receivers. Both the receivers show approximately the same power reception capabilities. However, as compared to single transmitter- receiver power transfer efficiency, the 1 transmitter to 2 receiver efficiency sees an increment from 51.4 $\%$ to 66 $\%$.

Table ST 4 – Two receiver power transfer and loss ($watts$) across 8 $meters$ of metal sheet

Transmitted	Rx1	Rx2	Loss
40	13.15	13.25	13.6

Table ST 5 – Two receiver power transfer and loss ($watts$) across 15 $meters$ of metal sheet

Transmitted	Rx1	Rx2	Loss
40	4.78	4.05	31.17

The table ST 5 shows the multi receiver capability at 15 m, the overall efficiency is 22%. Beyond this range, the power transfer drops below 10%. With proper optimization of thickness of coils and spacing, one can obtain higher values of power transfer range.

5.2 Effect on other devices in vicinity

In the previous sections it has been pointed out that, other devices in vicinity have to be of electrically comparable lengths, apart from being resonant. A Samsung Galaxy Note 5 mobile phone was used as a test device (with an inbuilt wireless charging receiver unit, tuned at 13.3MHz). The mobile phone was kept on the metal sheet, 25 $Watts$ of power was fed into the transmitter side at 13.3MHz and 27 MHz. The wireless receiver unit of the phone was unable to pick the power. It was found that the phone's functioning was normal, with normal touch screen response times and internet access. See the video in the supporting information. Two more test subjects were chosen, which included a table lamp with an LED bulb and a 13 $inch$ laptop (laptop was kept in ON status). Both the test devices were subjected to 50 $watts$ of transmitter power. None of the test devices showed any abnormal behavior.

5.3 Effect of metal enclosure

The table ST 6 summarizes the power transfer metrics comparison between open air and the box type partial enclosure power transfer metrics of the proposed system. The open air multi receiver efficiency is around 66 % at 8 m , while the enclosed case efficiency is 64.5 % for the same distance. The metal enclosure experimental result, clearly indicates that

Table ST 6 – Partial metal shielded power transfer metrics to two receivers. Transmitted power 40 $watts$ along 8 m metal

	Rx1	Rx2
Open	13.15	13.25
Partial Box Enclosure	13.12	12.68

the helical coil counterpoise plays no role in the power transmission through radiation. As claimed earlier, the helical coil counterpoise drives the receiver terminals to a high voltage. Therefore, the proposed system is non-radiative [16, 17, 18, 19, 20].

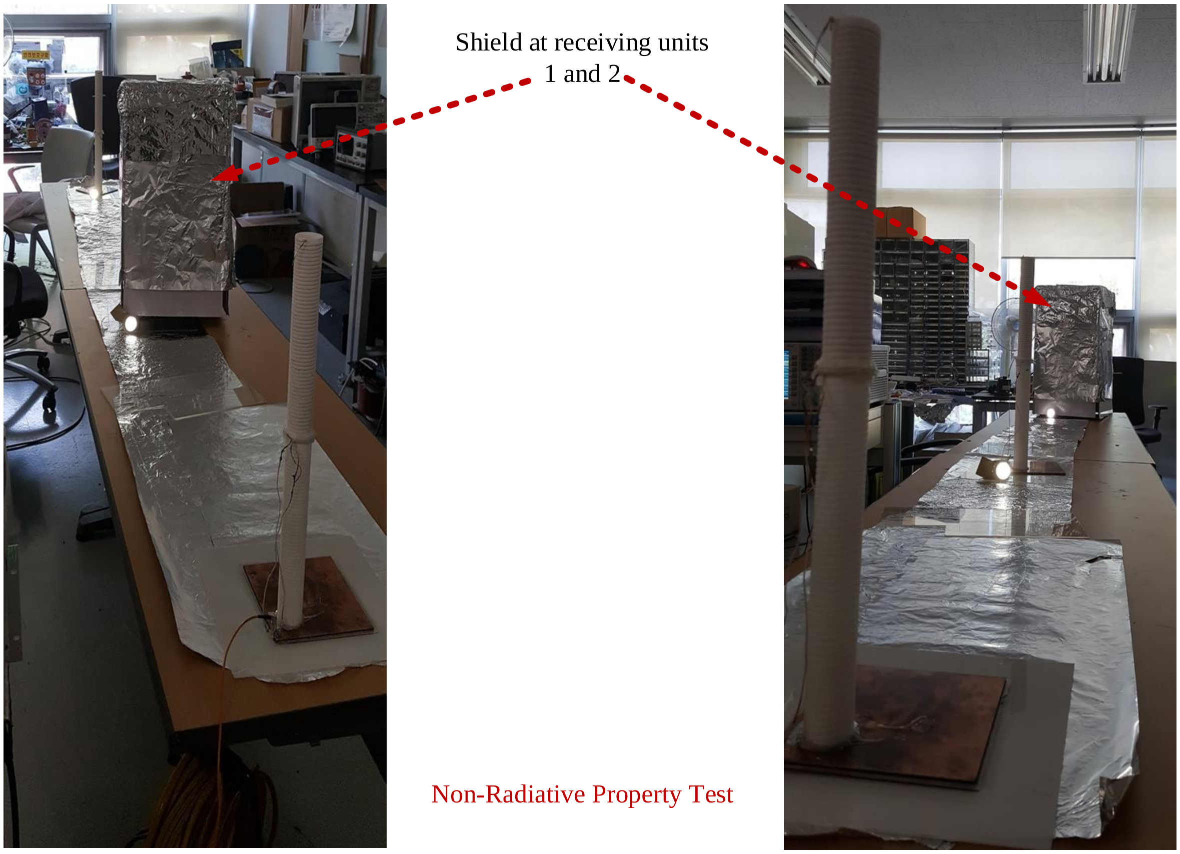

Fig. S 12 – Shields deployed at receiving units 1 and 2.

6 Power Demo

https://youtu.be/UOc42vG9Vhs

7 Metal Neutral

https://youtu.be/6dqdgtNcEQI

8 Electronic devices in vicinity

https://youtu.be/7UPf7M1EiiE

9 Multi Receiver

https://youtu.be/qRQKoeCV-8k

10 Tx in The Metal Enclosure

https://youtu.be/t-_HAqxcnkI

11 Background of Wave Based Approaches

We will briefly take a note of other μ-wave approaches, which are either under development or have been commercialized.

The wave based approaches can be broadly classified according to their applications-energy harvesting and transmission-reception. The former case lead to the development of the concept of rectenna(rectifier-antenna). The later case saw the usage of magnetron, as most of the said systems were developed in the frequency regime of 2.45 to 5.8 GHz. The antenna of a rectenna system receives the electromagnetic energy emanating from the surrounding systems, e.g. stray WIFI or mobile phone communication signals. The high frequency rectifier circuit converts the incoming wave and directs the rectified current to charge the batteries or load[23, 24, 25].

Table ST 7, a qualitative and quantitative comparison is done for the various ubiquitous systems utilizing MPT. Most of the applications are in the mW range and the frequency

Table ST 7 – MPT and Ubiquitous: Quantitative and Qualitative Comparison

Article	Input Power	Output Power	Observations and Comments
[23]	60 mW	39 mW	65 $\%$ efficiency achieved in milliwatts Not useful for high power applications
[24]	10 KW	5 kW	50 $\%$ efficiency 1.2 meters range No comment on misalignment issue
[25]	35 dBm or 3.16W	3 dBm or 2mW	0.063 $\%$ efficiency in any case the maximum power received by Rx is very less
[26]	27 dBm or 0.5W	-10 dBm or 1μW	Threshold limit was set to 1μW as per regulations In any case efficiency 0.00027$\%$
[27]	39 dBm or 7.94W	22.02 dBm or 0.16 W	Efficiency 2$\%$ received current of 40 mA, not suitable for fast charging
[28]	220 W	DC-RF max 75 $\%$	No information on received RF power

under question is 2.45 and 5.8 GHz. Besides the efficiency and power handling capability is far below the presented ZW system. However, one does find an exception in the form of [24], where the power handling capability is 10 kW. The presented ZW system has thick copper components and litz wire. The ZW system in its present form can easily handle upto $3kW$. The dielectric materials can be replaced by air to increase the power handling capabilities to 20 kW and beyond.

As listed in table ST 8, the same issue of extremely low power handling continues. These kind of systems can not handle anything beyond 5 watts of power. There is one similarity between Noda & Shinoda's work and the presented ZW wave concept. Both these systems are capable of handling multi receivers. This is essentially due to the fact that the said systems do not exhibit strong coupling.

As listed in table ST 9, MPT has been utilized for robots inside metallic pipelines. The initial attempts seemed to have failed due to losses arising due to multipath issues created due to the network of pipelines. This is an understandable problem. Its important to have

Table ST 8 – 2D Sheet: Quantitative and Qualitative Comparison

Article	Input Power	Output Power	Observations and Comments
[29]	500 mW	52.4-37.2 mW	Sum of 8 receivers total efficiency 87%, range was 5 mm receivers never leave the sheet
[30]	30.3 dBm or 1 W	24.2 dBm or 0.263 W	26.3% efficiency receiver never leaves the sheet.
[31]	-	-	Median efficiency 31% Follow-up of [29, 30] Low power system (mW)
[32]	2 W	80mW	4 % efficiency, a preceding article of [29, 30, 31].

Table ST 9 – MPT in pipes: Quantitative and Qualitative Comparison

Article	Input Power	Output Power	Observations and Comments
[33],[34]	-	-	Initial attempts to charge a robot system ran into problems due to uncertainties arising in multi-pipe transmission of radiowaves
[35]-[37]	-	-	100 mW range, very low power

a loosely guided wave in these scenarios and hence, the ZW system can easily resolve this issue. The key is to go beyond 6 GHz for the ZW system.

As per the articles [35]-[37] the issue is the low power handling capabilities. The article does not mention, how they are going to charge a 10 kW electric vehicle using 100 mW received power.

The published articles [38, 39] pertain to use of ventilator ducts as waveguides. The ZW system does not need such a closed boundary conditions. Besides, the power handling capabilities of ZW are comparable to the listed systems in table ST 10, the efficiency of the concept prototype of the ZW system performs at par with the listed systems.

By this time, this is absolutely clear that the proposed ZW system can not be com-

Table ST 10 – MPT in buildings: Quantitative and Qualitative Comparison

Article	Input Power	Output Power	Observations and Comments
[38],[39]	-	-	52 % efficiency, a practical system. However, in open metal sheets this system efficiency will degrade to less than 10%

pared with through air transmission system. Simply because, the ZW system works at an interface of two media air-metal. Therefore, it is a futile attempt to even compare with the MPT systems for charging unmanned aerial vehicles (UAV), listed in table ST 11.

Table ST 11 – MPT UAV's: Quantitative and Qualitative Comparison

Article	Input Power	Output Power	Observations and Comments
SHARP system	-	-	The literature available in public domain does not mention power transmission the applications are limited to communications. Moreover, this is a far-field system
[40]	5 W	-	Max received power 65mW far-field system

Listed in table ST 12 is the MPT using beam forming techniques. This is a totally different concept and in its published form, can not be applied to dynamic vehicle charging. However, in far-field region, this technique would be useful [41, 42]. In the published articles listed in the table, the vehicles are being charged when they are stationary. Once again, ZW system has distinct advantages over this kind of a system, since ZW is a propagating wave at the interface. Therefore, a single metal line can run across the highway and the mobile vehicle can extract power from the metal line. Other dynamic charging systems employ copper coils embedded in the roads, e.g. OLEV system from KAIST, Korea.

In conclusion, the proposed ZW system is based on a completely different concept, frequency regime and application. It would not be appropriate to draw a comparison among the various wave based systems. Also, we would be indulging in a great disservice to the research community and technologies by claiming that ZW system is ”superior”. In a fair unbiased light, ZW system has a unique set of properties and have a unique set of applications. Same is the case for MPT based systems.

Table ST 12 – MPT EV's: Quantitative and Qualitative Comparison

Article	Input Power	Output Power	Observations and Comments
[41]	-	-	This is a beam forming concept.
[42]	-	-	Beam forming concept.

12 On Iso-Phases and Iso-Amplitudes

Zenneck in his 1907 paper mentions about relationship of vertical electric field and horizontal electric field and the angle of tilt of the Iso-Amplitudes. The relationship for E-fields in the Air,(the Y and Z axis is defines as per the Fig S13):

$$\left|\frac{E_{oy}}{E_{oz}}\right| = \sqrt{\frac{q_0}{\sqrt{1+q^2}}} \tag{S28}$$

where, $q_0 = \nu\epsilon_0/\sigma$ and $q = \nu\epsilon/\sigma$. Where, ν is the wave number, ϵ_0 is free space permitivitty ,ϵ is relative permitivitty and σ is conductivity. For conductors the relationships were given as:

$$\left|\frac{E_y}{E_z}\right| = \frac{1}{\frac{E_{oy}}{E_{oz}}} \tag{S29}$$

Zenneck showed the angle relationship using the elliptical diagram as shown in Fig. S 13a as:

$$\frac{OB}{OA} = \left|\frac{E_y}{E_z}\right|, \frac{OA_1}{OA} = \frac{OB_1}{OB} = sin\phi \tag{S30}$$

As per Zenneck, drawing only half the ellipse diagram each in air and conductive surface the Fig. S 13 b occurs when,$\left|\frac{E_y}{E_z}\right|$ if very small, then $\phi_0 = 1$. The Fig. S 13 c when $\left|\frac{E_y}{E_z}\right|$ not small, then ϕ_0 is very small. The Fig. S 13 d when $\left|\frac{E_y}{E_z}\right|$ and ϕ_0 not small. Where, $\phi = 90° - \phi_0$.

Zenneck in his article considers a dielectric constant of 80 for the conductive material and the conductivity was taken 10^{-12} to 10^{-13} in C.G.S units. Which happens to be a case of ***Lossy Dielectric***. Hence, the forward tilting mode is observed in his analysis.

12.1 Material properties and Tilt?

As evident, for a Lossy dielectric the tilt of modes is forward or in the direction of the propagation. For the case of dielectric with higher conductivity the tilt angle is lower as

compared to lossy dielectric at a carefully selected conductivity and dielectric constant the tilt would be zero as is the case shown in Fig. 13 b, this also is the case for Surface waves. Therefore, a pure or efficient surface wave mode is excited at inductive impedance structures or corrugated metal surfaces of comparable wavelengths.

But, when the conductive surface is a noble metal such as aluminium, then the conductive $\sigma = 3.8 \times 10^{-7} S/m$. As per the drude's model the real part of complex permitivitty is negative. However, the imaginary part which is controlled by the σ becomes dominant in the MHz regime[43].

Therefore, in equation S29; by substituting ϵ with$\epsilon^{'} - j\epsilon^{''}$ we get:

$$\left|\frac{E_y}{E_z}\right| = \left(\sqrt{\frac{\nu\sigma\epsilon_0}{\sigma^2 + \nu^2(\epsilon' - j\epsilon'')^2}}\right)^{-1} \tag{S31}$$

The result of the equation S29 therefore becomes extremely small. For example for aluminium the calculated ϕ_0 would be $r = |z| = 0.02451; angle = argz = 106.82$, which implies the corresponding $\phi = 90 - 106.82 = -16.82°$, the negative value of angle is the backward tilt. Likewise, for a lossy dielectric with the constants $\epsilon_r = 4.1$, $\sigma = 3.8 \times 10^{-3} S/m$, loss tangent of 0.58 at 27 MHz, $r = |z| = 0; angle = argz = -4.08563$ the calculated $\phi_0 = -4.8°$, therefore the corresponding $\phi = 90 - (-4.0856) = 94.08°$, hence a forward tilt. These results have been validated using ANSYS HFSS simulation in Fig. S14.

The conductivity of metals ranges in the order of 10^7 - silver: $6.3 \times 10^7 S/m$; copper: $5.8 \times 10^7 S/m$; gold: $5.96 \times 10^7 S/m$; Iron: $1 \times 10^7 S/m$. The excited Zenneck wave would witness a backward tilt. We derived the equation S29 from the original equations presented by Zenneck(S28 and S29)

To test Zenneck's theory, the simulation of Iso-amplitudes and their tilt angles is listed in Fig. S 13

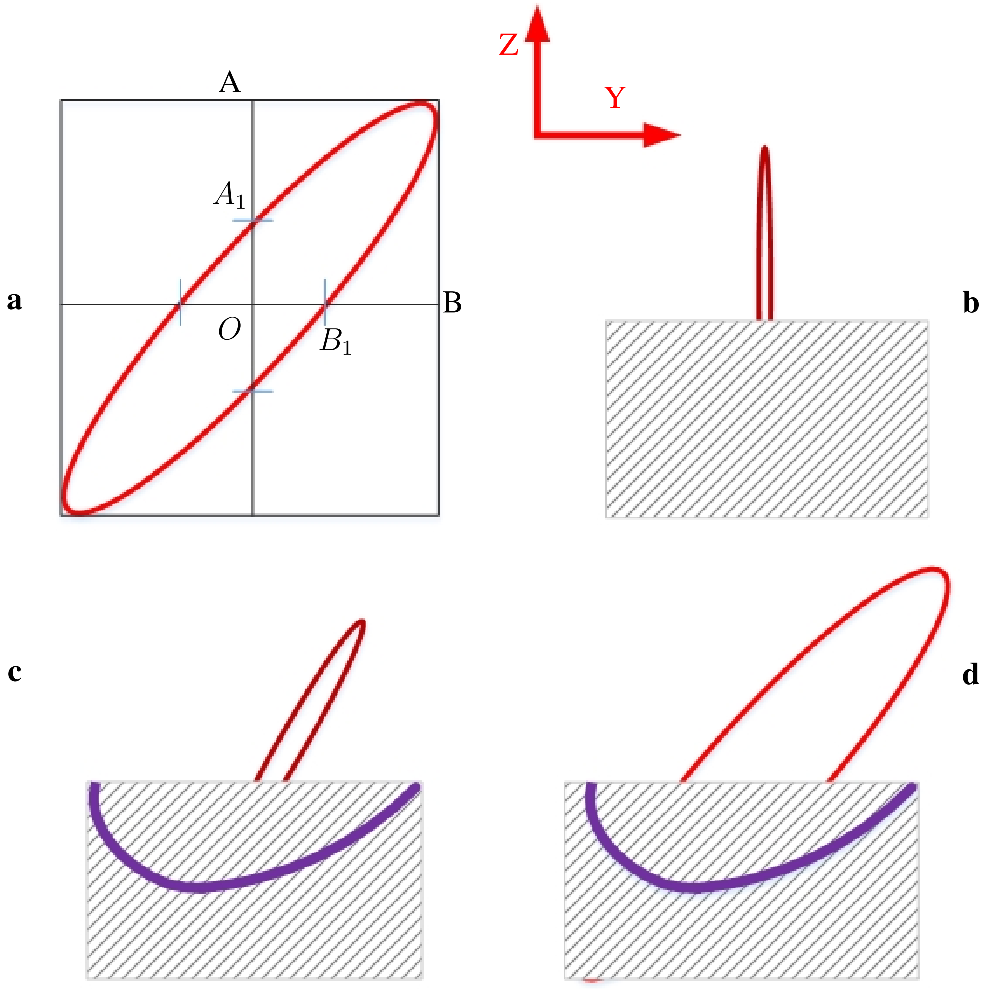

Fig. S 13 – Angle of tilt defined by Zenneck in 1907 article

a

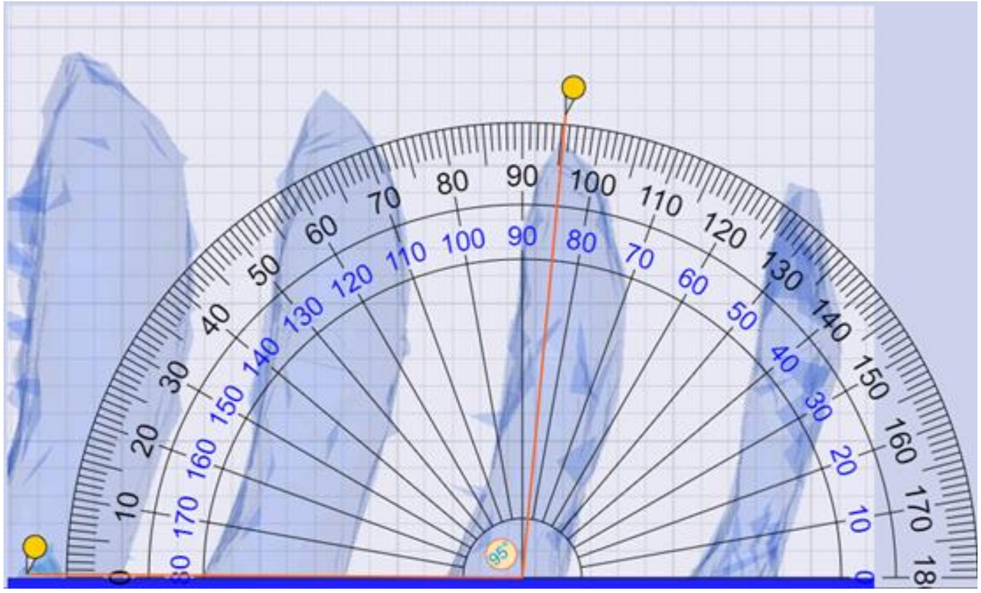

$\phi = 95°$, where $\phi = 90° - \phi_0$

b

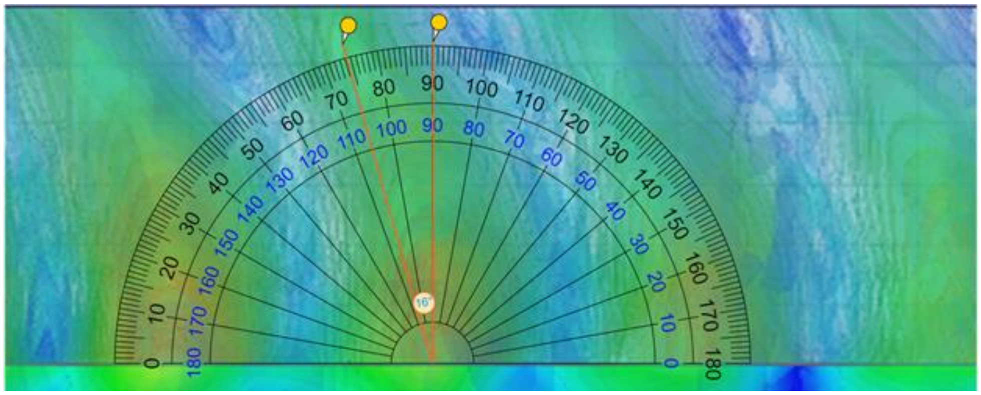

$\phi = -16°$, where $\phi = 90° - \phi_0$

Fig. S 14 – ANSYS HFSS Simulation Validation of Equation 1, Tilt angle according to media properties. (a) Lossy dielectric the angle of tilt is ϕ_0=-4.8; so ϕ=94.8 (b) For Metal the tilt angle is ϕ_0=106 and ϕ=90-(106)= -16;

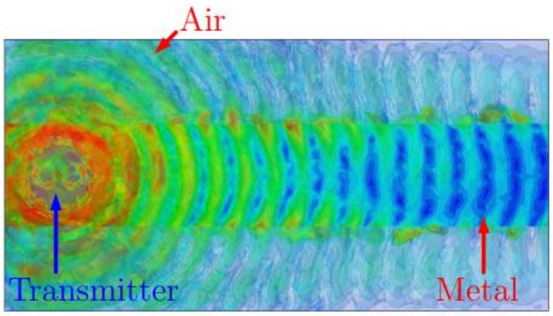

Fig. S 15 – ANSYS HFSS Simulation Validation of Equation 1, Tilt angle according to media properties. (a) Lossy dielectric the angle of tilt is ϕ_0=-4.8; so ϕ=94.8 (b) For Metal the tilt angle is ϕ_0=106 and ϕ=90-(106)= -16;

References

[1] C.A.Balanis, Antenna Theory: Analysis and Design (John Wiley & Sons, NY, 2005).

[2] S.R. Best, *URSI Radio Science Bulletin* **2016**, 13(2016).

[3] R.W. Ziolkowski, *IEEE Antennas and Wireless Propagation Letters* **7**, 581-584(2008).

[4] J. Malik, S.K. Oruganti, S. Song, N.Y. Ko, and F.Bien, *Appl. Phys. Lett.* **112**, 234102(2018).

[5] C.W. Van Neste, R. Hull, J.E. Hawk, A. Phani and T. Thundat, *Cambridge Wireless Power Journal*, **3**, 117-125(2016).

[6] C.W. Van Neste, J.E. Hawk, A. Phani, J.A.J Backs, R. Hull,T. Abraham, S.J. Glassford, A.K. Pickering, and T. Thundat, *Cambridge Wireless Power Journal* **1**, 75-82(2014).

[7] S. Schelkunoff, *IRE Trans. on Antenna and Propagation* **7**, 133-139 (1959).

[8] T. K. Sarkar, M. N. Abdallah, M. Salazar-Palma and W.M. Dyab, *IEEE Antennas and Propagation Magazine* **59**, 77-93(2017).

[9] T.K. Sarkar, W.M. Dyab, M.N. Abdallah, M. Salazar-Palma, M.V.S.N. Prasad and S. Ting, *IEEE Trans. on Antennas and Propagation* **62**, 4162-4170 (2014).

[10] H.M. Barlow, and A.L. Cullen, *Proceed. of the IEE - Part III: Radio and Comm. Engineering* **100**, 329-341 (1953).

[11] A. Kurs, A. Karalis, R. Moffatt, J.D. Joannopoulos, P. Fisher, M. Soljacic, *Science* **317**, 83-86 (2007).

[12] J. Li, X. Huang, C. Chen, L. Tan, W. Wang W. and J. Guo, *AIP Advances* **7**, 056675 (2017).

[13] F. Jangal, N. Bourey, M. Darces, F. Issac and M. Hélier, *Hindawi, International J. of Antennas and Propagation* **2016**,1 (2016).

[14] A. Sommerfeld, *Ann. d. Phys.* **28**, 665-736(1909).

[15] K.A.Michalski and J.R. Mosig, *Journ. of Electromagnetic Waves and Applications* **30**,1-42,2016.

[16] S.K. Oruganti, and F. Bien *IEEE Wireless Power Transfer Conference (WPTC)*, 247(2014).

[17] S.K. Oruganti, S.H. Heo, H. Ma, and F. Bien, *Electron. Lett.* **50**, 886(2014).

[18] S.K. Oruganti, O. Kaiyrakhmet and F. Bien, *URSI Asia-Pacific Radio Science Conference*, 318(2016).

[19] S.K. Oruganti, S.H. Heo, H. Ma, and F. Bien, *IEEE Sensors Journal* **15**, 2062(2015).

[20] S.K. Oruganti, Thesis, *Ulsan National Institute of Science and Technology*, 2016.

[21] A. Ahlbom, et al. *Health Phys.* **74**, 494(1998)

[22] Maehara, D., Akai, R., Tran, G.K., Sakaguchi, K., Sampei, S., Araki, K., & Iwai, H., Experiment on battery-less sensor activation via multi-point wireless energy transmission, *IEEE* 24th Annual International Symposium on Personal, Indoor, and Mobile Radio Communications, London, 2336-2340, (2013).

[23] Furukawa, M., T. Minegishi, T. Ogawa, Y. Sato, P. Wang, H. Tonomura, M. Teramoto, and N. Shinohara, Wireless Power Transmission to 10kW Output 2.4 GHz-band Rectenna Array for Electric Trucks Application (in Japanese), *IEICE Technical Report*, WPT2012-7, 36-39, 2013.

[24] Sakaguchi, K., R.P. Wicaksono, K. Mizutani, and T. Khanh, Wireless Grid to Realize Ubiquitous Networks with Wireless Energy Supply (in Japanese), *IEICE Tech. Report*, **109**, 442.

[25] Ichihara, T., T. Mitani, and N. Shinohara, Study on Intermittent Microwave Power Transmission to a ZigBee Device, *IEEE MTT-S* International Microwave Workshop Series on Innovative Wireless Power Transmission: Technologies, Systems, and Applications, 209-212, 2012.

[26] Sakaguchi, K., R.P. Wicaksono, K. Mizutani, and T. Khanh, Wireless Grid to Realize Ubiquitous Networks with Wireless Energy Supply (in Japanese), *IEICE Tech. Report*, **109**, 442.

[27] Hashimoto, K., T. Ishikawa, T. Mitani, and N. Shinohara, Improvement of a ubiquitous power source, **Proc. of International Union of Radio Science (URSI) General Assembly**, 2011.

[28] Mitani, T., H. Yamakawa, N. Shinohara, K. Hashimoto, S. Kawasaki, F. Takahashi, H. Yonekura, T. Hirano, T. Fujiwara, K. Nagano, H. Ueda, and M. Ando, Demonstration Experiment of Microwave Power and Information Transmission from an Airship, Proc. of 2nd International Symposium on Radio System and Space Plasma 2010, pp. 157-160, 2010.

[29] Noda, A. and Shinoda,H., Selective Wireless Power Transmission Through High-QFlat Waveguide-Ring Resonator on 2-D Waveguide Sheet, *IEEE Transactions on Microwave Theory and Techniques* **59**, 2158-2167, (2011).

[30] Noda, A. and Shinoda,H., Waveguide-ring resonator coupler with class-F rectifier for 2-D waveguide power transmission, *IEEE MTT-S International Microwave Workshop*,259-262, 2012.
doi: 10.1109/IMWS.2012.6215806

[31] Noda, A. and Shinoda,H., Antinull 2-D Waveguide Power Transfer Based on Standing Wave Diversity, *IEEE Transactions on Microwave Theory and Techniques*, **66**, 306-318, 2018.

[32] Shinoda, H., Y. Makino, N. Yamahira, and H. Itai, Surface sensor network using inductive signal transmission layer, *Int. Conf. on Networked Sensing Systems (INSS)* 201-206, 2007.

[33] Hirayama, K., N. Shinohara, K. Hashimoto, H. Matsumoto, T. Nakauchi, and Y. Yoshida, "Fundamental Study of Microwave Power Transmission to a Robot Moving in Gas Pipes (in Japanese)", Proc. of IEICE Comm., p. 116, March 1997.

[34] Hirayama, K., N. Shinohara, H. Matsumoto, and I. Nagano, "Study of Microwave Power Transmission to a Robot Moving in Gas Pipes (in Japanese)", Proc. of IEICE Comm., p. 25, March 1999.

[35] Ishino, S., A. Kishimoto, Y. Takimoto, Y. Arai, Y. Huang, and N. Shinohara, "Study on WPT System Using a Radio Wave Hose as a New Transmission Line", Proc. of 2015 IEEE Wireless Power Transfer Conference (WPTc2015), P4.1, May 2015.

[36] Ishino, S., T. Miyagawa, and N. Shinohara, "A New Slotted Waveguide Using a Resin Hose with Metallic Coating", Proc. of 2015 Asia-Pacific Microwave Conference (APMC), Dec. 2015.

[37] Shibata, T., Y. Aoki, M. Otsuka, T. Idogaki, and T. Hattori, "Microwave Energy Transmission System for Microrobot", IEICE Trans. Electr., Vol.E80-C, No. 2, pp. 303-308, 1997.

[38] Shinohara, N., N. Niwa, K. Takagi, K. Hamamoto, S. Ujigawa, J.-P. Ao, and Y. Ohno, "Microwave Building as an Application of Wireless Power Transfer", Wireless Power Transfer, pp. 1-9, 2014.4.

[39] Takahashi, K., J.-P. Ao, Y. Ikawa, C.-Y. Hu, H. Kawai, N. Shinohara, N. Niwa, and Y. Ohno, "GaN Schottky Diodes for Microwave Power Rectification", Japanese Journal of Applied Physics (JJAP), Vol. 48, No. 4, pp. 04C095-1 - 04C095-4, 2009.

[40] Dunbar, S., F. Wenzl, C. Hack, R. Hafeza, H. Esfeer, F. Defay, S. Prothin, D. Bajon, and Z. Popovic, Wireless Far-Field Charging of a Micro-UAV, *IEEE Wireless Power Transfer Conference*, T1.2, May 2015.

[41] Shinohara, N., "Beam Efficiency of Wireless Power Transmission via Radio Waves from Short Range to Long Range", Journal of the Korean Institute of Electromagnetic Engineering and Science, Vol. 10, No. 4, pp. 224-230, 2011.

[42] Shinohara, N., "Wireless Power Transmission Progress for Electric Vehicle in Japan", Proc. of 2013 IEEE Radio & Wireless Symposium (RWS), pp. 109-111, 2013.

[43] Sergeichev,K.F., Karfidov,D.M., Andreev, S. E., Sizov, Yu. E., & Zhukov, V.I. Excitation and Propagation of Sommerfeld–Zenneck Surface Waves on a Conducting Strip in the Centimeter-Wave Band. *Journal of Comm. Tech. and Electronics*, *63*,326,2018.
